# CaY@C_2*n*_: Exploring Molecular Qubits with Ca–Y Metal–Metal Bonds

**DOI:** 10.1021/jacs.4c04720

**Published:** 2024-08-21

**Authors:** Jiawei Qiu, Laura Abella, Xiya Du, Zhengkai Cao, Zhiwen He, Qingyu Meng, Yingjing Yan, Josep M. Poblet, Lei Sun, Antonio Rodríguez-Fortea, Ning Chen

**Affiliations:** † College of Chemistry, Chemical Engineering, and Materials Science and State Key Laboratory of Radiation Medicine and Protection, Soochow University, Suzhou, Jiangsu 215123, P.R. China; ‡ Departament de Química Física i Inorgànica, Universitat Rovira i Virgili, Marcel·lí Domingo 1, Tarragona 43007, Spain; § Department of Chemistry, School of Science and Research Center for Industries of the Future, Westlake University, Hangzhou, Zhejiang Province 310030, China; ∥ Institute of Natural Sciences, Westlake Institute for Advanced Study, Hangzhou, Zhejiang Province 310024, China; ⊥ Key Laboratory for Quantum Materials of Zhejiang Province, Department of Physics, School of Science, Westlake University, Hangzhou, Zhejiang Province 310030, China

## Abstract

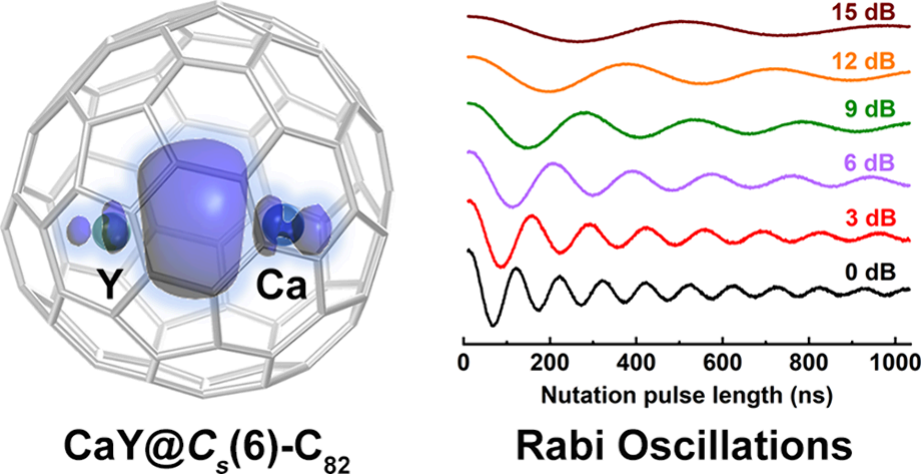

Metal–metal bonding is crucial in chemistry for advancing our understanding of the fundamental aspects of chemical bonds. Metal–metal bonds based on alkaline-earth (Ae) elements, especially the heavier Ae elements (Ca, Sr, and Ba), are rarely reported due to their high electropositivity. Herein, we report two heteronuclear di-EMFs CaY@*C*_*s*_(6)-C_82_ and CaY@*C*_2*v*_(5)-C_80_, which contain unprecedented single-electron Ca–Y metal–metal bonds. These compounds were characterized by single-crystal X-ray crystallography, electron paramagnetic resonance (EPR) spectroscopy, and DFT calculations. The crystallographic study of CaY@*C*_*s*_(6)-C_82_ shows that Ca and Y are successfully encapsulated into the carbon cage with a Ca–Y distance of 3.691 Å. The CW-EPR study of both CaY@*C*_*s*_(6)-C_82_ and CaY@*C*_2*v*_(5)-C_80_ exhibits a doublet, suggesting the presence of an unpaired electron located between Ca and Y. The combined experimental and theoretical results confirm the presence of a Ca–Y single-electron metal–metal bond with substantial covalent interaction, attributed to significant overlap between the 4s4p orbitals of Ca and the 5s5p4d orbitals of Y. Furthermore, pulse EPR spectroscopy was used to investigate the quantum coherence of the electron spin within this bond. The unpaired electron, characterized by its s orbital nature, is effectively protected by the carbon cage, resulting in efficient suppression of both spin–lattice relaxation and decoherence. CaY@*C*_*s*_(6)-C_82_ behaves as an electron spin qubit, displaying a maximum decoherence time of 7.74 μs at 40 K. This study reveals an unprecedented Ae–rare-earth metal–metal bond stabilized by the fullerene cages and elucidates the molecular qubit properties stemming from their unique bonding character, highlighting their potential in quantum information processing applications.

## Introduction

Metal–metal bonds are of great significance in expanding our understanding of the nature of chemical bonding.^1−3^ Over the past two decades, a number of remarkable discoveries like Zn–Zn,^4^ Cr–Cr,^5^ Mg–Mg,^6^ and Be–Be^7^ bonds, have been reported, attracting great attention to metal–metal bonding. To date, the studies of metal–metal bonding are mainly focusing on the main^8^ and transition group^9^ elements. Metal–metal bonds based on alkaline-earth (Ae) elements, especially the heavier Ae elements (Ca, Sr, and Ba), are rarely reported due to their high electropositivity. In this case, only a few examples of metal–metal bonds involving Ae metals have been reported so far. All of these bonds are formed between Ae and main-group^1,10−14^ or transition metals^1,15−17^ with the exception of Be–Be and Mg–Mg bonds. In particular, the formation of metal–metal bonds between Ae metals and rare-earth (Re) metals is challenging with conventional synthetic methods due to the high electropositivity of both Ae and Re metals. To the best of our knowledge, this kind of metal–metal bond has not been reported to date.

Metal atoms can be encapsulated into the internal cavities of fullerenes to form endohedral metallofullerenes (EMFs), which are stabilized by transferring electrons from the metals to the carbon cages. Di-EMFs, i.e., carbon cages encapsulating two metal atoms, are considered as an ideal model for investigating metal–metal bonding. On the one hand, carbon cages can protect metal dimers from external influences. On the other hand, the Coulomb repulsion between the metal ions can be limited by the confinement effect of the carbon cages, which can shorten the distance between the metals and thus facilitate the formation of metal–metal bonds.^18^ Indeed, recent studies have indicated that direct metal–metal bonds can be formed between the metal ions encapsulated in di-EMFs, such as M_2_@C_82_ (M_2_ = Sc_2_,^19^ Y_2_,^18^ Er_2_,^20^ Lu_2_,^21^ and ScY^22^) and M_2_@C_80_ (M_2_ = U_2_^23^ and Th_2_^24^).

In particular, single-electron metal–metal bonds can also be obtained in the form of dimetallofullerene derivatives^25−30^ or azafullerenes.^31−34^ With the encapsulated [Ln-e-Ln] bonding motifs (Ln = Dy, Gd, and Tb), these di-EMFs exhibit excellent magnetic properties.^29^ Such special properties of single-electron metal–metal bonds have also been found in organometallic chemistry. Recently, Gould et al. reported the dilanthanide complexes (Cp^iPr5^)_2_Ln_2_I_3_ with single-electron Ln-Ln bonds (Ln = Gd, Tb, or Dy), which displayed the highest 100 s blocking temperatures among all reported single-molecule magnets up to date.^35^

Recently, we reported a series of mixed-valence di-EMFs with single-electron actinide-lanthanide metal–metal bonds, namely, ThDy@C_2*n*_ (2*n* = 72, 76, 78, and 80) and ThY@C_2*n*_ (2*n* = 72 and 78).^36^ Surprisingly, these di-EMFs are stable in their pristine form and do not require any derivatization. A similar case was reported by Yang et al., who synthesized the endofullerene LaTi@C_2_*_n_*.^37^ Both fullerene families share a common feature: the two encapsulated metal atoms have an odd sum of valence electrons, which appears to facilitate the formation of the single-electron metal–metal bonds inside the pristine fullerene cages. This inspires us to explore the possibility of an Ae–Re metal–metal bond, in which alkaline-earth elements possess two valence electrons and rare-earth elements possess three valence electrons.

Herein, we report the successful synthesis and characterizations of Ca-based heteronuclear di-EMFs, i.e., CaY@*C*_*s*_(6)-C_82_ and CaY@*C*_2*v*_(5)-C_80_. These novel compounds were characterized by single-crystal X-ray crystallography, UV–vis–NIR spectroscopy, electron paramagnetic resonance (EPR) spectroscopy, and theoretical calculations. We identified the formation of an unprecedented Ca–Y single-electron metal–metal bond inside a carbon cage. Moreover, the electron in this bond exhibits significant spin coherence at relatively high temperatures, behaving as a qubit with potential utility for quantum information science.

## Results and Discussion

CaY@C_2*n*_ (2*n* = 80 and 82) were synthesized by a modified Krätschmer–Huffman DC arc discharge method.^38^ In brief, 0.33 g of CaO, 0.67 g of Y_2_O_3_, and 2.13 g of graphite powder (molar ratio of Ca:Y:C = 1:1:30) were packed in each graphite rod (6.7 g, without filling). About 300 graphite rods were vaporized in the arcing chamber under a 200 Torr He atmosphere. The resulting carbon soot was extracted by CS_2_ for 24 h. Then, a multiple-step HPLC procedure was employed to isolate and purify CaY@C_2*n*_ (2*n* = 80 and 82). The purity of CaY@C_2*n*_ (2*n* = 80 and 82) was confirmed by the single peaks in the HPLC chromatogram and matrix-assisted laser desorption/ionization time-of-flight mass spectrometry (MALDI-TOF-MS). The MALDI-TOF-MS spectra of CaY@C_82_ and CaY@C_80_ show single peaks at *m*/*z* = 1112.898 and 1088.856, respectively, and their isotopic distributions agree well with the calculated ones (Figure S3). The estimated yields of CaY@C_80_ and CaY@C_82_ are ca. 0.20 and 0.22 mg, respectively.

**Figure 1:**
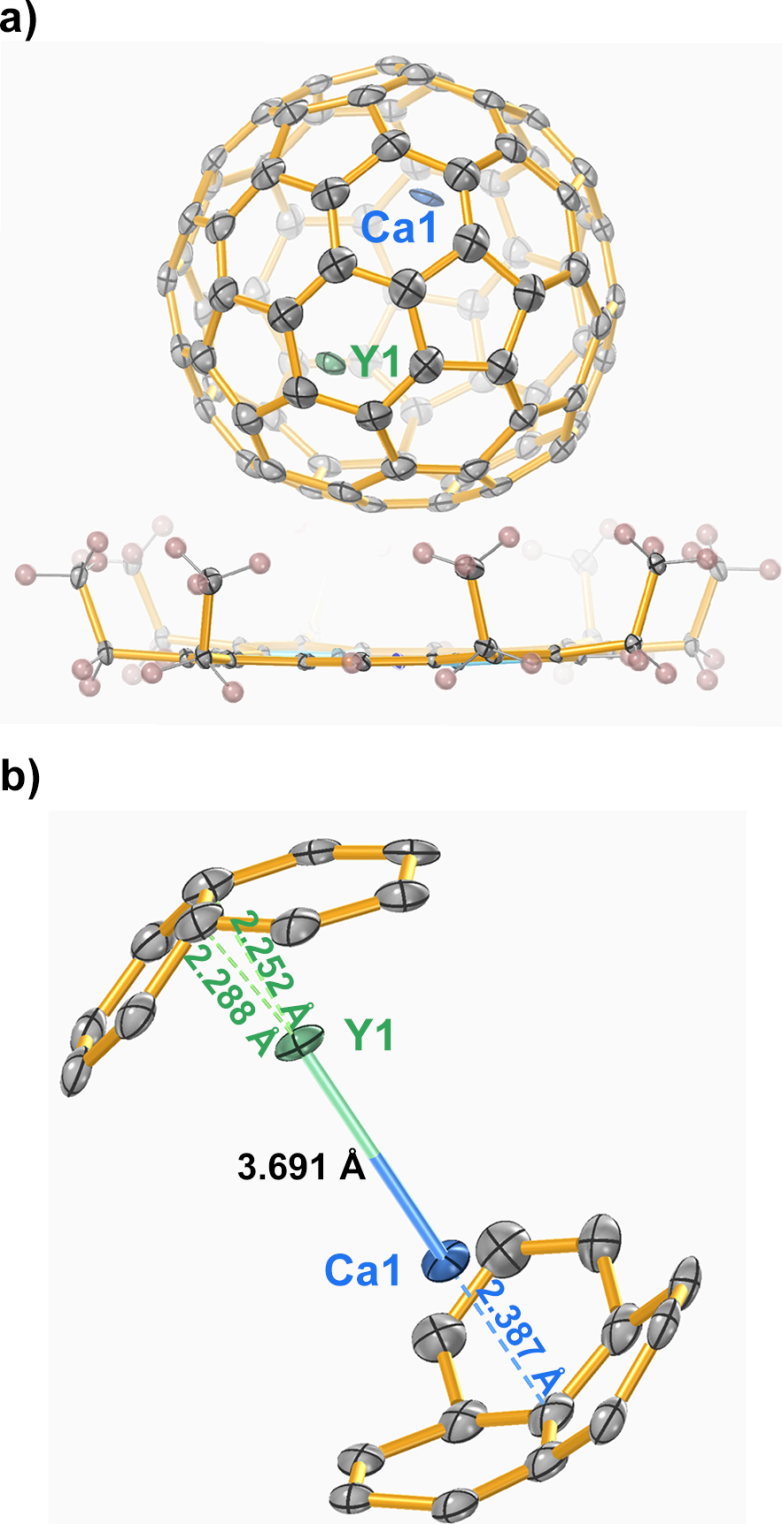
(a) ORTEP drawing for CaY@*C*_*s*_(6)-C_82_·[Ni^II^(OEP)] with 15% thermal ellipsoids. Only one cage orientation and the major Ca and Y sites are shown. For clarity, the solvent molecule and minor metal sites are omitted. (b) Detailed structure of the major Ca and Y sites interacting with the closest fragments of the *C*_*s*_(6)-C_82_ cage.

Ca-based heteronuclear di-EMFs have rarely been reported before. The only study of the synthesis of CaHo@C_82_ was reported in 2007.^39^ However, lacking characterization and quantum-chemical studies, the molecular and electronic structure of this compound has never been identified. In this study, black block cocrystals of CaY@C_82_·[Ni^II^(OEP)]·2C_6_H_6_ (OEP = 2,3,7,8,12,13,17,18-octaethylporphyrin anion) were obtained by slow diffusion from a benzene solution of Ni^II^(OEP) into a CS_2_ solution of CaY@C_82_. The molecular structure of CaY@C_82_ was unambiguously determined by single-crystal X-ray diffraction and refined as CaY@*C*_*s*_(6)-C_82_, which was monoclinic with the *C*2/*m* space group (Figure 1).

Due to the crystallographic mirror plane of the *C*2/*m* space group, the carbon cage of CaY@*C*_*s*_(6)-C_82_ has two equivalent orientations with the same occupancy of 0.5. The metal atoms inside the carbon cage exhibit some degree of disorder. Ca1 and Y1 were identified as the major sites for Ca and Y atoms, respectively, with an occupancy of 0.3157(17) for both. Ca1_m_ and Y1_m_ are generated from Ca1 and Y1 through the crystallographic mirror plane. The minor sites, namely, Ca2, Ca3, Y2, and Y3, are all located on the crystallographic mirror plane. Ca2 and Y2 have the same occupancy (0.172(3)) as well as for Ca3 and Y3 (0.196(3)). For clarity, only one orientation of the carbon cage and the major sites of internal metal atoms, i.e., Ca1 and Y1, are selected for further analysis.

Similar to the position of Y3 in Y_2_@*C*_*s*_(6)-C_82_,^18^ Y1 resides over the [6,6] carbon bond with the shortest Y cage distances ranging from 2.252 to 2.288 Å, which is in good agreement with the density functional theory (DFT) calculations (2.345–2.357 Å, Figure S6). This Y cage distance is slightly shorter than that in Y_2_@*C*_*s*_(6)-C_82_ (ranges from 2.323 to 2.331 Å in the X-ray structure and from 2.407 to 2.421 Å at the DFT level), which suggests that the Y cage interaction is enhanced by replacing the other Y atom with a Ca atom. Furthermore, when a Y atom is replaced with a Ca atom, the position of the respective metal atom within the carbon cage is altered. Different from Y5 in Y_2_@*C*_*s*_(6)-C_82_, which is located near the [5,6] bond, Ca1 in CaY@*C*_*s*_(6)-C_82_ is located at the junction of the three hexagons with the Ca cage shortest distance of 2.387 Å. A similar phenomenon has been observed when a Sc atom replaces a Y atom in Y_2_@*C*_3*v*_(8)-C_82_.^22^ The distance of Ca1–Y1 is determined to be 3.691 Å (vs 3.705 Å in calculations), which is comparable to the Y–Y distance in Y_2_@*C*_*s*_(6)-C_82_ (3.635 Å vs 3.613 Å in calc.)^18^ and the Sc–Y distance in ScY@*C*_3*v*_(8)-C_82_ (3.674 Å).^22^ Considering the presence of metal–metal bonds in both Y_2_@*C*_*s*_(6)-C_82_ and ScY@*C*_3*v*_(8)-C_82_, this Ca–Y distance might indicate that there is a bonding interaction between Ca and Y.

The electronic features of CaY@C_2*n*_ (2*n* = 80 and 82) were studied by UV–vis–NIR spectroscopy (Figure S5). The spectrum of CaY@*C*_*s*_(6)-C_82_ exhibits absorption peaks at 765, 828, and 1190 nm, which are similar to those of M_2_@*C*_*s*_(6)-C_82_ (M = Lu,^21^ Y,^18^ and Er^20^). It is well-known that the UV–vis–NIR spectra of EMFs with the same isomer structure and formal charge state of the carbon cage are almost identical due to π→π* transitions of the cage.^40^ Consequently, this UV–vis–NIR spectrum suggests that CaY@*C*_*s*_(6)-C_82_ has the same electronic configuration with M_2_@*C*_*s*_(6)-C_82_ (M = Lu, Y, and Er), namely, (CaY)^4+^@C_82_^4–^. The spectrum of CaY@C_80_ exhibits a major absorption peak at 672 nm, which is similar to the one observed in the spectrum of Sc_2_O@*C*_2*v*_(5)-C_80_.^41^ This suggests that the *C*_2*v*_(5)-C_80_ isomer is the same as the cage isomer of CaY@C_80_, which can be formally described as (CaY)^4+^@C_80_^4–^.

To analyze the electronic structure and the Ca–Y bonding interaction, DFT calculations at the PBE0/TZP/D3^42−48^ level were performed for CaY@C_2*n*_ (2*n* = 80 and 82) cages, showing a spin-doublet ground electronic state for both of them (see computational details for more information). Different positions of Ca and Y atoms within the *C*_2*v*_(5)-C_80_ and *C*_*s*_(6)-C_82_ cages were computed to analyze the most likely location of the metals inside the cages (see Tables S3 and S4 and Figures S8 and S9). Our calculations show that the lowest-energy structure of CaY@*C*_*s*_(6)-C_82_ corresponds to the one observed in the X-ray structure. Other positions of the metals show a relative energy of 3.5 kcal·mol^–1^ (orientation B in Table S4 and Figure S9) and around 9 kcal mol^–1^ (C–E), which confirm the somewhat degree of disorder found in experiments. DFT optimizations of the crystallographic Ca2–Y2 and Ca3–Y3 positions evolve to orientation C (Figure S14). For CaY@*C*_2*v*_(5)-C_80_, the lowest-energy arrangement of the metals corresponds to their positioning analogous to the sites occupied by Sc atoms in Sc_2_O@*C*_2*v*_(5)-C_80_.^41^ If the metal atoms exchange their positions, the relative energy increases to 4.2 kcal·mol^–1^. Other positions of the metal atoms are found at higher energies (>12.2 kcal·mol^–1^).

**Figure 2:**
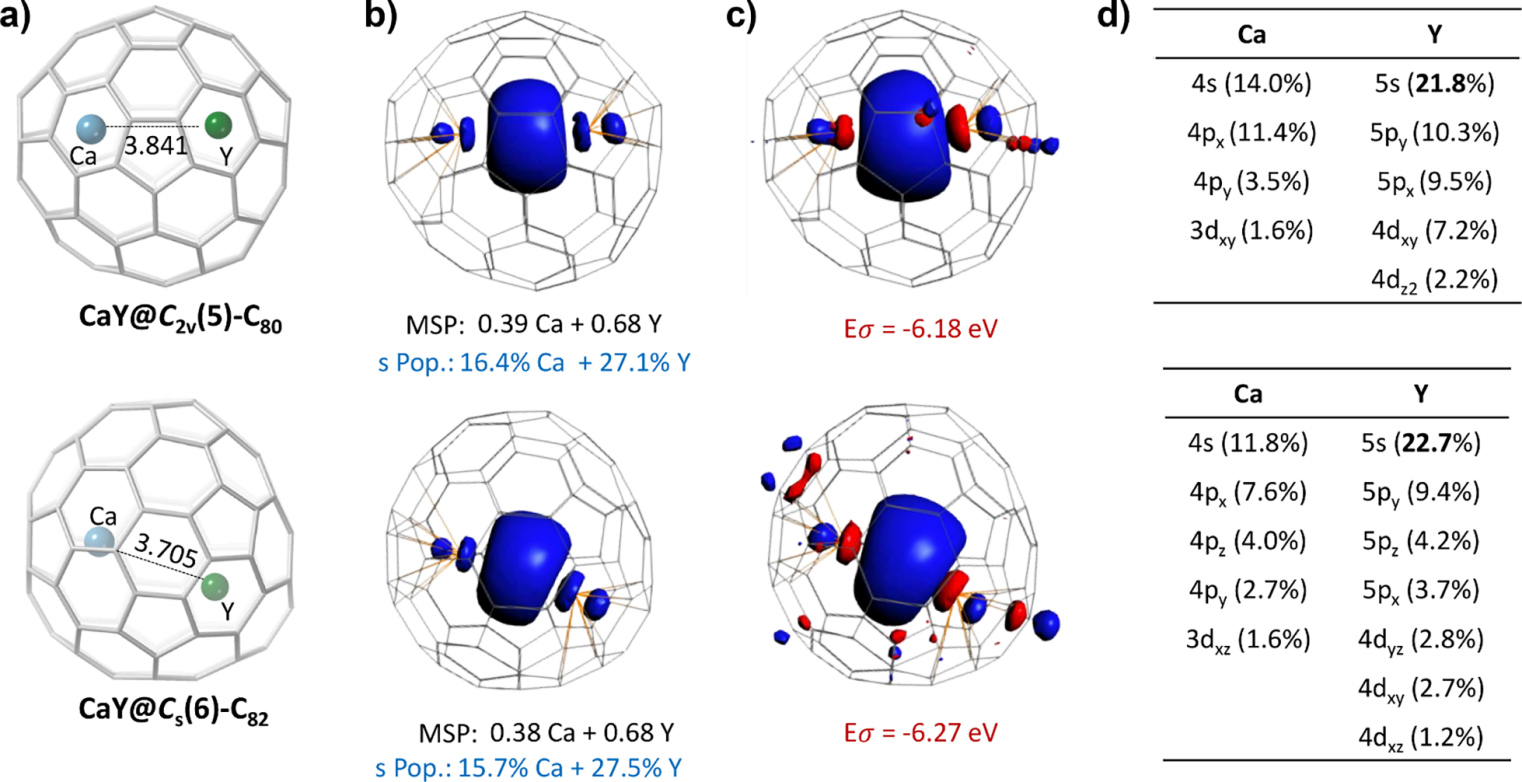
For CaY@*C*_2*v*_(5)-C_80_ (top) and CaY@*C*_*s*_(6)-C_82_ (bottom); (a) DFT-optimized structure, where the Ca–Y distance (in Å) is indicated. (b) Spin density distribution with an isosurface of ±0.002 au, Mulliken spin populations (MSP), and amount of s spin density population (s Pop.) of the metals. (c) Molecular orbital (MO) isosurface (±0.03 au) for the delocalized σ orbital *a*_1_ for the α-spin with the corresponding MO energy (in eV). (d) Molecular orbital contributions of the σ-type bonding orbital formed essentially by *n*s, *n*p, and (*n –* 1)d metal orbitals.

The following description corresponds to the lowest-energy geometries of CaY@*C*_2*v*_(5)-C_80_ and CaY@*C*_*s*_(6)-C_82_ (Figure 2). The optimized Ca–Y distances are 3.841 and 3.705 Å for CaY@*C*_2*v*_(5)-C_80_ and CaY@*C*_*s*_(6)-C_82_, respectively, resulting in the latter to be very close to experiments (3.691 Å). For CaY@*C*_2*v*_(5)-C_80_, the Ca and Y are both located at [6,6] bonds of pyracylene motifs, showing that the Y metal–cage distances (2.357 Å) are closer than those of Ca (2.473 Å) (Figure S7). In CaY@*C*_*s*_(6)-C_82_, the Ca is located at the center of a hexagon from an s-indacene motif with the closest metal–cage distance of 2.490 Å (vs 2.387 Å in experiments), while the Y is placed on top of a [6,6] bond of a pyracylene motif with metal–cage distances of 2.345–2.357 Å (Figure S6), slightly larger than those from experiments (2.252–2.288 Å). The position of Y in a pyracylene motif in CaY@*C*_2*v*_(5)-C_80_ and CaY@*C*_*s*_(6)-C_82_ is in accordance to the largest molecular orbital contribution of the LUMO+1 of neutral *C*_2*v*_(5)-C_80_ and *C*_*s*_(6)-C_82_ cages, as well as to the most negative region of the potential electrostatic maps of *C*_2*v*_(5)-C_80_^4–^ and *C*_*s*_(6)-C_82_^4–^ anions (see Figures S10 and S11, respectively).

Figure 2b shows the spin density distributions for CaY@C_2*n*_, indicating that the unpaired electron is delocalized between the two metal atoms. Additionally, Figure 2c illustrates that the unpaired electron resides in an *a*_1_ sigma-type orbital, which results from a significant overlap between the 4s4p orbitals of Ca and 5s5p4d of Y (Figure 2d). Despite the fairly symmetric appearance of the spin density distribution and the sigma *a*_1_ orbital representation, the atomic Mulliken spin populations of approximately 0.4 e for Ca and 0.7 e for Y indicate that the metal–metal bond displays a certain degree of polarization. This polarization is primarily driven by the s-type orbitals, with associated s spin density populations of around 16% for Ca and 27% for Y.

These data indicate that the formal oxidation states would be close to 1.5+ for Ca and 2.5+ for Y, if the unpaired electrons were equally shared. Thus, there is an electron transfer of four electrons from the CaY cluster to the C_2*n*_ cage, resulting in an electronic structure of (CaY)^4+^@(C_2*n*_)^4–^ according to the ionic model. The molecular orbital (MO) diagrams for the ground spin-doublet state of CaY@*C*_2*v*_(5)-C_80_ and CaY@*C*_*s*_(6)-C_82_ are provided in the Supporting Information (SI). As mentioned, a delocalized sigma-bonding orbital *a*_1_ is observed in both CaY@C_2*n*_ cages (Figure 2c) with the energy depending on the metal–metal bond length. Specifically, the shorter the Ca–Y distance (3.841 vs 3.705 Å), the lower the energy of the σ-bonding orbital (−6.18 vs −6.27 eV, respectively). In addition, both oxidation and reduction^49^ of CaY@*C*_*s*_(6)-C_82_ are predicted to take place on the carbon cage, with the one-electron Ca–Y σ-bond remaining essentially unaltered (Figure S15).

Two notable features distinguish this novel Ae–Ln bond from the recently detected one-electron σ bond in ThLn@C_2*n*_ (Ln = Y and Dy).^36^ (i) It exhibits some degree of polarization due to the larger electronegativity difference between Y (Pauling, 1.22) and Ca (1.00). (ii) There is minimal involvement of the 3d orbitals from Ca (Figure 2d). Additionally, this direct Ca–Y interaction clearly demonstrates that under specific conditions, the Ca orbitals can indeed participate in covalent interactions.

To further characterize the Ae(Ca)–Ln(Y) bond interaction, we performed the Bader’s quantum theory of atoms in molecules^50^ analysis. Bader postulated the bond critical point (bcp) between two atoms as a necessary and sufficient condition for the atoms to be bonded. The corresponding values of the electron density (ρ) and the Laplacian of the electron density (∇^2^ρ) at the bcp for CaY@*C*_2*v*_(5)-C_80_ and CaY@*C*_*s*_(6)-C_82_ are provided in Table 1, which verify the presence of an accumulation of charge density in the center of the metal–metal bond. The electron density values of both CaY@C_2*n*_ systems closely resemble those of other reported systems, such as ThY@*D*_3*h*_(5)-C_78_,^36^ Y_2_@C_80_-CH_2_Ph,^27^ and Y_2_@C_79_N^31^ as well as the hypothetical SrY@*C*_2*v*_(5)-C_80_ and SrY@*C*_*s*_(6)-C_82_ cages (see Table 1). It is worth noting that CaY@C_2*n*_ systems present slightly lower electron density values compared to the corresponding SrY@C_2*n*_ endohedral fullerenes, which show smaller metal–metal distances. All these systems present a negative sign in the Laplacian of the electron density; however, M–Y (M = Ca and Sr) systems show lower absolute values than for the other calculated EMFs. Larger absolute values of ∇^2^ρ are found for Ca–Y compared to Sr–Y systems, consistent with the fact that the Sr–Y σ bonds are somewhat more polarized. Interestingly, the CaY@C_2*n*_ systems exhibit the largest ρ values among other experimental reported Ca–X (X = Al, Sn, Co, and Fe) complexes.^10,14,16,51^ Therefore, for instance, a Ca–Fe bond with a bond length of 2.98 Å^51^ displays a charge density of 0.022 e/Å^3^, considerably lower than that observed for Ca–Y, even though the latter has a significantly longer bond length (Table 1). Furthermore, the Ca–Y bond shows a negative sign on the ∇^2^ρ, consistent with the Ca–Y covalent interaction deduced from the singly occupied delocalized σ orbital. The Ca–Y bonds characterized in this study can be considered as the bonds with the highest degree of covalent character involving a calcium atom reported to date.

**Table 1:** Computed EPR and Electronic and Structural Parameters for Different XY@C_2*n*_ Systems^g^

	CaY*@C*_2*v*_(5)-C_80_	SrY@*C*_2*v*_(5)-C_80_	CaY@*C*_*s*_(6)-C_82_	SrY@*C*_*s*_(6)-C_82_	ThY@*D*_3*h*_(5)-C_78_	Y_2_@C_80_-CH_2_Ph	Y_2_@C_79_N
*g*-value	1.983 (1.983)	1.972	1.979 (1.982)	1.969	1.803 (1.825)	1.978	1.975 (1.974)
*A*^a^	236 (252)	266	245 (260)	275	206 (200)	204 (224)	203 (224)
*d*(Y–X)^b^	3.841	3.750	3.704 (3.147)	3.657	4.137 (4.144)	3.879	3.863
spin Y^c^	0.68	0.72	0.68	0.73	0.59	0.53	0.53
spin X^c^	0.39	0.34	0.38	0.34	0.43	0.52	0.52
s Pop.^d^	27.1	30.0	27.5	30.7	22.4	21.4	21.3
ε_σ_^LUMO^^e^	–3.50	–3.34	–3.59	–3.38	–3.68	–3.95	–4.01
ρ_bcp_^f^	0.091	0.106	0.100	0.112	0.107	0.108	0.109
∇^2^ρ_bcp_^f^	–0.063	–0.027	–0.026	–0.015	–0.194	–0.204	–0.201

a Hyperfine coupling (in MHz).

b Metal–metal distance (in Å).

c Atomic Mulliken spin densities for Y and X.

d Amount of the s spin density population on Y (in %).

e Energies of the sigma LUMO (beta) orbital (in eV).

f Electron density and Laplacian of the electron density at the bond critical points are given in [e Å^–3^] and [e Å^–5^], respectively.

g Experimental values are in parentheses.

**Figure 3:**
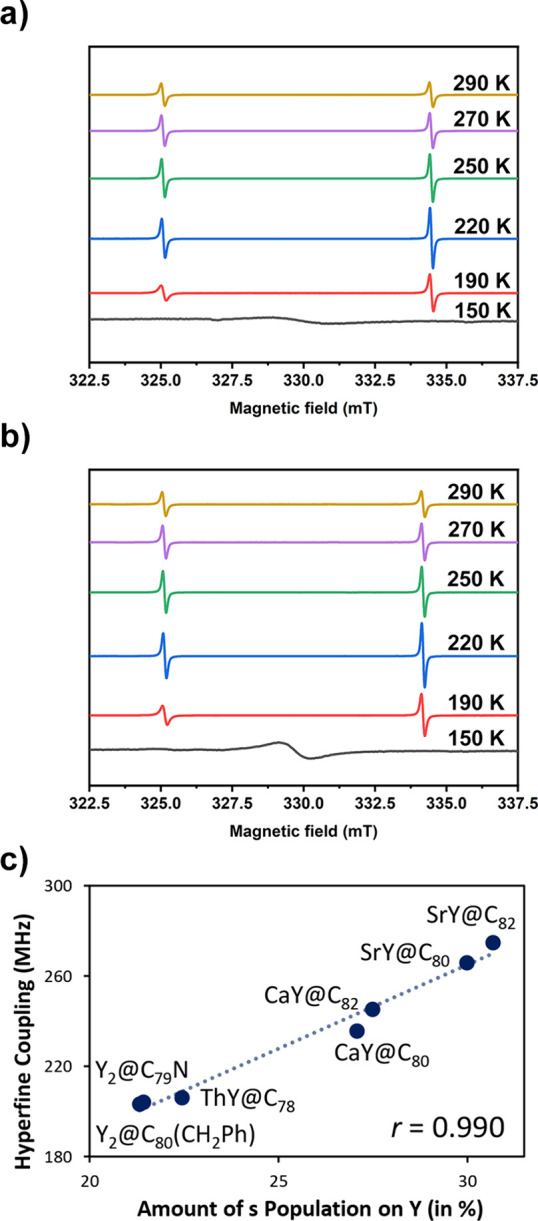
EPR X-band spectra of CaY@*C*_*s*_(6)-C_82_ (a) and CaY@*C*_2*v*_(5)-C_80_ (b) measured in CS_2_ solution at 290, 270, 250, 220, 190, and 150 K. (c) Representation of the correlation between the hyperfine coupling constant (in MHz) and the amount of the s spin density population located on the Y atom (in %) for all the XY@C_2*n*_ systems shown in Table 1. The correlation coefficient *r* is also given.

Continuous-wave (CW) EPR spectroscopy experiments were also performed on CaY@C_2*n*_ (2*n* = 80 and 82) to obtain further information on their open-shell electronic structure. As is shown in Figure 3, the EPR spectra of purified CaY@C_2*n*_ (2*n* = 80 and 82) in a CS_2_ solvent show, for each compound, a doublet at 290, 270, 250, 220, and 190 K, which indicates hyperfine coupling between an unpaired electron and an ^89^Y nucleus (nuclear spin I = 1/2 of ^89^Y, 100% natural abundance, while the nuclear spin of ^40^Ca is 0 in 96.941% natural abundance). As the temperature decreases, the high magnetic field signal becomes increasingly more dominant compared to the low magnetic field signal. Such a kind of paramagnetic anisotropy results from insufficient rotational averaging due to the restricted motion of the Y nucleus and the unpaired electron.^52^ At 150 K, the hyperfine structure disappears, and the spectrum exhibits a single broad peak since the nonglassy CS_2_ solvent freezes at this temperature, causing molecular aggregation where spin–spin dipolar interaction dominates over the hyperfine interaction. Fitting of the EPR spectra collected at 290 K revealed that the hyperfine coupling constants (abbreviated as hfcc or *A* below) of CaY@*C*_*s*_(6)-C_82_ and CaY@*C*_2*v*_(5)-C_80_ are 260 and 252 MHz, respectively (Figure S16). These hfcc constants are significantly larger than those found for Y@C_82_ whose electron spin mainly resides on the carbon cage.^53^ Thus, the strong hyperfine coupling in CaY@*C*_*s*_(6)-C_82_ and CaY@*C*_2*v*_(5)-C_80_ indicates that their unpaired electrons are mainly metal-centered, as confirmed by the spin density distribution (Figure 2b).^54^ The Lorentzian line shape of EPR spectra indicates negligible hyperfine coupling from ^13^C, which is consistent with the result obtained from pulse EPR characterization (*vide infra*). The isotropic *g*-values of CaY@*C*_*s*_(6)-C_82_ and CaY@*C*_2*v*_(5)-C_80_ are 1.9819 and 1.9827, respectively, which are comparable to those of Y_2_@C_79_N (*g* = 1.9740)^31^ and Y_2_@C_80_(CH_2_Ph) (*g* = 1.9733), as well as their spin densities.^27^ Thus, EPR experiments also indicate the formation of a single-electron metal–metal bond between Ca and Y.

DFT calculations are in full agreement with experiments (Table 1). The calculated *g*-values for CaY@*C*_2*v*_(5)-C_80_ and CaY@*C*_*s*_(6)-C_82_ are 1.983 and 1.979, respectively, nearly identical with the experimental values. The calculated hfcc are 236 MHz for CaY@*C*_2*v*_(5)-C_80_ and 245 MHz for CaY@*C*_*s*_(6)-C_82_, in good agreement with the experimental values (vs 252 and 260 MHz, respectively). The spin density located on the Y atom is the same for both CaY@C_2*n*_ cages (0.68 e). The slightly larger hfcc in CaY@*C*_*s*_(6)-C_82_ than in CaY@*C*_2*v*_(5)-C_80_ is most likely related to the larger amount of the s spin density population located on yttrium (27.5 vs 27.1%). These data are consistent with the electronic structure, as they indicate that the unpaired electron is confined to yttrium and delocalized on calcium, suggesting the formation of a polarized single-electron bond between Y and Ca atoms.

To further explore the electronic structure and EPR parameters on CaY@C_2*n*_ systems, a computational study involving other XY@C_2*n*_ families was performed. Table 1 provides the most relevant data from this DFT study. We have worked with some previously reported systems ThY@*D*_3*h*_(5)-C_78_,^36^ Y_2_@C_80_(CH_2_Ph),^27^ and Y_2_@C_79_N,^31^ as well as with the hypothetical SrY@C_2*n*_ (2*n* = 80 and 82) family, where the Ca is replaced with a Sr atom in the corresponding CaY@C_2*n*_ (2*n* = 80 and 82) cages. Our calculations reproduce very well the experimental *g*-values and the hfcc of all of the systems analyzed herein, as seen in Table 1. ThY@*D*_3h_(5)-C_78_ shows the lowest *g*-value among these systems, and its hfcc is significantly lower than those of CaY@C_2*n*_ cages but very similar to Y_2_@C_80_(CH_2_Ph) and Y_2_@C_79_N. This fact is related to the spin density of Y as well as the amount of the s spin density population located on the Y atom. For ThY@*D*_3*h*_(5)-C_78_, Y_2_@C_80_(CH_2_Ph), and Y_2_@C_79_N, all of these values are very similar. CaY@C_2*n*_ cages show larger values of Mulliken spin density on Y (0.68 vs ∼0.59) and a larger amount of the s population on Y (27% vs ∼22%). Therefore, larger values of hfcc are observed in the CaY@C_2*n*_ cages. For the SrY@C_2*n*_ (2*n* = 80 and 82) family, although similar *g*-values are found, this hypothetical family displays the largest hfcc (266 MHz for SrY@*C*_2*v*_(5)-C_80_ and 275 MHz for SrY@*C*_*s*_(6)-C_82_), which is also in line with the larger values of Mulliken spin density of Y (∼0.72 e) and the amount of the s spin density population on Y (∼30%). Interestingly, a good correlation exists between the hfcc and the amount of the s population on the Y atom (Figure 3c). A reasonably acceptable correlation is still found if hfcc values are plotted with respect to the total spin population on Y (see Figure S13). We note that all of these systems present a two-center single-electron σ bond.

The demonstration of single-electron bonds in CaY@C_2*n*_ inspired us to investigate their potential as electron spin qubits. A spin qubit should possess a long spin–lattice relaxation time (*T*_1_) and decoherence time (*T*_2_) at relatively high temperatures (above 77 K) to facilitate its implementation in quantum information technologies.^55^ As revealed by the DFT calculations (Table 1), the partially distributed electrons on both Ca and Y exhibit significant s orbital character. Because the s electron is free of orbital angular momentum, its spin–orbit coupling is naturally quenched, giving rise to slow spin–lattice relaxation.^56,57^ Since *T*_1_ serves as the upper limit to *T*_2_, *T*_2,max_ = 2*T*_1_, the electron spin with s orbital character likely maintains quantum coherence at relatively high temperatures. Such an effect has manifested itself in an Y^2+^-based coordination complex, [K(2,2,2-cryptand)][Y(C_5_H_4_SiMe_3_)_3_], whose ^2^*S*_1/2_-like ground state shows microsecond-scale *T*_1_ and *T*_2_ at room temperature.^56^ In addition, the carbon cage provides a rigid, isolated, and almost nuclear spin-free environment to the endohedral electron spin, which suppresses spin–lattice relaxation and decoherence.^58^ As a result, fullerenes encompassing spin centers could exhibit an excellent qubit performance. For instance, Sc_3_C_2_@C_80_ maintains coherence at room temperature in solution,^59^ Gd_2_@C_79_N behaves as a qudit with a large ground spin state (*S* = 15/2),^60^ and functionalized N@C_60_ displays an exceptionally long *T*_2_ (tens of microseconds at 77 K) and multiple addressable quantum states that enable quantum error correction and simultaneous operations of two quantum logic gates.^61^ It is also noteworthy that Y@C_82_ displays coherence up to 130 K albeit its electron spin distributes dominantly on the carbon cage.^62^ Putting together, the electron residing on the Ca–Y bond should display slow spin–lattice relaxation and maintain coherence at elevated temperatures.

**Figure 4:**
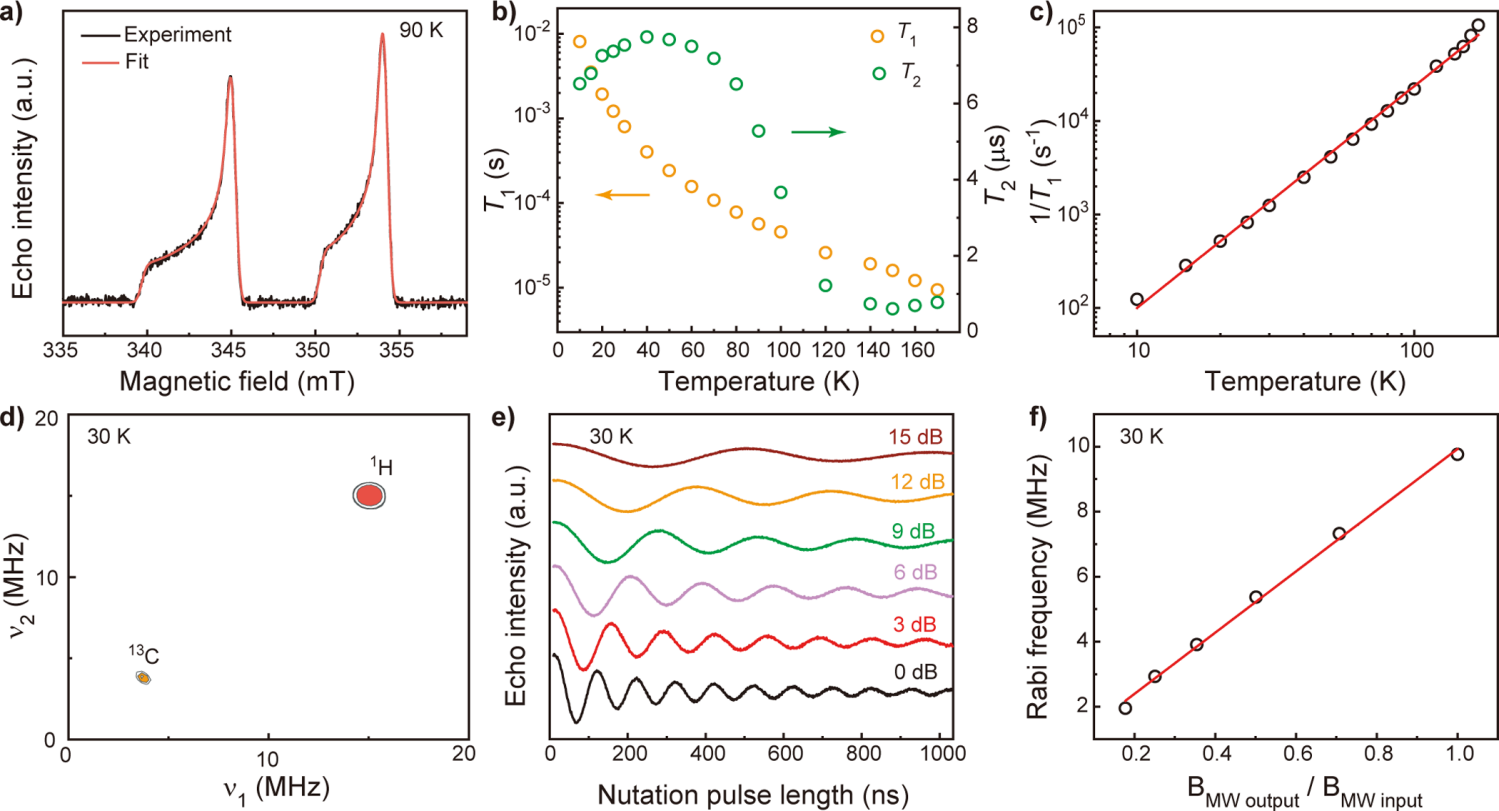
X-band pulse EPR of CaY@*C*_*s*_(6)-C_82_ dissolved in toluene. (a) Echo-detected field sweep (EDFS) spectrum at 90 K under a microwave frequency of 9.656 GHz. (b) Temperature dependence of *T*_1_ and *T*_2_. (c) Corresponding 1/*T*_1_ versus temperature. The red line is a fit to the data by the Raman relaxation process, , where *A*_Raman_ and *T* are the weighting coefficient and temperature, respectively. The fitted exponent *m* is 2.37. (d) HYSCORE spectrum collected at 30 K. (e) Rabi oscillations using various microwave attenuations. (f) Relationship between the Rabi frequency and *B*_MW output_/*B*_MW input_, where *B*_MW output_ and *B*_MW input_ represent the magnetic field of the output microwave and input microwave, respectively, the latter of which is equal to  (*A* represents the microwave attenuations in the unit of dB). The red line is a linear fit to the data.

We chose CaY@*C*_*s*_(6)-C_82_ as an example and characterized its electron spin dynamics by X-band pulse EPR spectroscopy. To avoid the molecular aggregation observed in the CS_2_ solution, we dissolved this compound in toluene, which is a glassy solvent. The echo-detected field sweep (EDFS) spectrum (Figure 4a) collected at 90 K shows features of *g*-anisotropy. Fitting of this spectrum revealed *g*_∥_ = 1.99948(6) and *g*_⊥_ = 1.97259(1) as well as anisotropic hyperfine splitting constants with *A*_∥_ = 296.1(5) MHz and *A*_⊥_ = 249.6(1) MHz. These values translate to isotropic *g*_iso_ = 1.98159 and *A*_iso_ = 266.0 MHz, which are consistent with the above-mentioned results of solution-phase CW-EPR and DFT calculations. The anisotropy and hyperfine coupling together give rise to four addressable EPR transitions, which likely exhibit comparable spin dynamics according to the previous study on [K(2,2,2-cryptand)][Y(C_5_H_4_SiMe_3_)_3_].^56^ Thus, we focused on the transition centered at approximately 354 mT in the following studies (the exact magnetic field varies with the microwave frequency used for the specific experiment).

We were able to acquire *T*_1_ and *T*_2_ of CaY@*C*_*s*_(6)-C_82_ from 10 to 170 K (Figures S17–S19 and Table S5); toluene melting and fast relaxation prevented measurements above 170 K. As shown in Figure 4b, *T*_1_ gradually decreases with an increasing temperature from 8.09 ms at 10 K to 9.5 μs at 170 K. The persistence of *T*_1_ up to 170 K is mainly attributed to the s orbital character of the Ca–Y single-electron bond as discussed above.^56^ The relaxation rate, 1/*T*_1_, is proportional to *T*^2.37^ (*T* represents temperature, Figure 4c), which may be attributed to the Raman relaxation mechanism in the high-temperature regime. When the experimental temperature is much higher than the Debye temperature (*T*_D_), i.e., *T* ≫ *T*_D_, all acoustic phonons are thermally accessible, giving rise to 1/*T*_1_ ∝ *T*^2^ behavior that is consistent with our experimental observation.^63,64^ This indicates that *T*_D_ ≪ 10 K, which is significantly lower than those of conventional inorganic solid-state materials that are typically hundreds of Kelvin. The exceptionally small *T*_D_ is likely the result of weak intramolecular interaction between the Ca–Y moiety and the C_82_ cage as well as weak intermolecular interaction between the C_82_ cage and toluene.^24,63,65^ Thus, the temperature dependence of *T*_1_ reinforces our hypothesis that the carbon cage protects the Ca–Y single-electron bond from the environment.

The coherence of the toluene solution of CaY@*C*_*s*_(6)-C_82_ is mainly limited by nuclear spin flip-flops and spin relaxation. The *T*_2_ is dramatically shorter than the *T*_1_ from 10 to 170 K, indicating that the dominant sources of decoherence are environmental nuclear spins. Under 40 K, the *T*_2_ gradually increases from 6.52 to 7.74 μs with an increasing temperature (Figure 4b). This phenomenon is likely associated with rotation of the methyl group in toluene, which facilitates the nuclear spin flip and reduces *T*_2_. Such enhancement of decoherence may be the most significant at a temperature lower than 10 K when the rotation resonates with the Larmor precession of nuclear spins, and it weakens at higher temperatures as a result of detuning, leading to a positive relationship between *T*_2_ and temperatures above 10 K.^62,64^ As the temperature increases above 40 K, the *T*_2_ first gradually decreases to 7.18 μs at 70 K. It then drops sharply above 70 K and reaches 1.22 μs at 120 K. These trends imply that the spin relaxation starts to limit coherence above 40 K.^62^ Finally, the *T*_2_ tends to level off above the glass temperature of toluene (*T*_glass_ = 113 K), and it becomes unmeasurable above the melting point of toluene (*T*_melt_ = 178.2 K). With the quantum coherence at 170 K, CaY@*C*_*s*_(6)-C_82_ joins Sc@C_82_, Y@C_82_, La@C_82_, Sc_2_@C_80_(CH_2_Ph), and Sc_3_C_2_@C_80_ to form a subclass of high-temperature EMF qubits that are compatible with a liquid nitrogen-cooled environment.^59,62,66^ Another subclass, e.g., Eu@C_2*n*_ (2*n* = 74, 80, 82, and 84), Gd@C_82_, animated Gd@C_82_, and Gd_2_@C_79_N, displays coherence at much lower temperatures (below 20 K) likely due to the f orbital character of their unpaired electrons.^60,67,68^ Their operation requires liquid-helium cooling or other sophisticated cryogenic technologies.

To gain a deeper understanding of the spin decoherence mechanism, we studied the nuclear spin bath of CaY@*C*_*s*_(6)-C_82_ by hyperfine spectroscopy. Specifically, we conducted remotely detected combination-peak electron spin echo envelope modulation (CP-ESEEM) experiments at 30 K (Figure S20a).^69,70^ This sequence probes the modulation of electron spin precession by nearby nuclear spins; therefore, it could reveal Larmor frequencies of nuclear spins and their hyperfine interactions with the electron spin. The CP-ESEEM spectrum displays two peaks located at 7.56 and 30.0 MHz (Figure S20b). They correspond to twice the Larmor frequencies of ^13^C and ^1^H nuclei, respectively. We further characterized details of hyperfine interactions by a remotely detected hyperfine sublevel correlation (HYSCORE) experiment conducted at 30 K. The HYSCORE spectrum displays two signals located at 3.75 and 15.0 MHz that are consistent with ^13^C and ^1^H nuclear spins, respectively. These signals do not exhibit apparent correlation ridges, indicating a negligibly weak hyperfine coupling (Figure 4d). Neither CP-ESEEM nor HYSCORE spectra show signals corresponding to ^89^Y, probably due to the strong hyperfine coupling of this nucleus, as revealed by the CW-EPR and EDFS measurements. As weakly coupled nuclear spins tend to behave as magnetic noises to undermine coherence, we speculate that ^13^C on the C_82_ cage and ^13^C and ^1^H in toluene are major sources of decoherence to CaY@*C*_*s*_(6)-C_82_. In contrast, ^89^Y resides within the nuclear diffusion barrier, so it has little contribution to decoherence.^71^ This indicates that deuteration of toluene may help improve *T*_2_, as demonstrated for Sc@C_82_, Y@C_82_, and La@C_82_.^62^ The *T*_2_ may be further improved by dynamic decoupling methods, such as the Carr–Purcell–Meiboom–Gill (CPMG) pulse sequence, as shown in Sc_2_@C_80_(CH_2_Ph) and Sc_3_C_2_@C_80_.^59,66^ These coherence enhancement strategies will be investigated in future studies.

To prove the ability of coherent manipulation of the electron spin in CaY@*C*_*s*_(6)-C_82_, we conducted nutation experiments at 30 K under various microwave powers. The nutation pulse rotates the electron spin on the Bloch sphere, with the rotation angle determined by the pulse length and microwave power, giving rise to iconic Rabi oscillations shown in Figure 4e. The fast Fourier transform (FFT) of the Rabi oscillation at each power reveals the corresponding Rabi frequency (Figure S21), which exhibits a linear dependence on the relative microwave magnetic field strength (*B*_MW output_/*B*_MW input_) (Figure 4f). This indicates that the electron spin of CaY@*C*_*s*_(6)-C_82_ behaves as a qubit that satisfies the Rabi relationship: ℏω_Rabi_ = *g*μ_Β_*SB*_1_, where ℏ is Planck's constant, ω_Rabi_ is the Rabi frequency, μ_Β_ is the Bohr magneton, and *B*_1_ is the microwave magnetic field strength. As the nutation pulse implements a single-qubit quantum logic gate, these experiments demonstrate the potential of CaY@*C*_*s*_(6)-C_82_ for quantum information science.

## Conclusions

In summary, unprecedented Ca–Y single-electron metal–metal bonds were formed inside heteronuclear di-EMFs, namely, CaY@*C*_*s*_(6)-C_82_ and CaY@*C*_2*v*_(5)-C_80_. The single-crystal X-ray crystallographic study unambiguously determined that the Ca ion and Y ion were encapsulated inside *C*_*s*_(6)-C_82_ with a Ca–Y distance of 3.691 Å. The UV–vis–NIR spectra as well as DFT computations suggest that four of the five valence electrons of the internal metals are formally transferred to the carbon cage. The CW-EPR study of both CaY@*C*_*s*_(6)-C_82_ and CaY@*C*_2*v*_(5)-C_80_ exhibits a doublet and implies that an unpaired electron is located between Ca and Y. The theoretical studies further confirm the existence of a Ca–Y single-electron metal–metal bond with substantial covalent interaction, attributed to significant overlap between the 4s4p orbitals of Ca and 5s5p4d orbitals of Y. With the s orbital character unpaired electron well-protected by the carbon cage, CaY@*C*_*s*_(6)-C_82_ behaves as an electron spin qubit, showcasing excellent coherence even at relatively high temperatures. Its potential applications in quantum information science warrant further exploration.

This study reveals the unexpected capacity of fullerenes to stabilize the Ae–Re bond, a target that remains challenging to achieve using conventional synthesis methods. Furthermore, it validates that by encapsulating two metal atoms with an odd sum of valence electrons, single-electron metal–metal bonds can be stabilized in the pristine fullerene cages. This paves the way for a novel method to achieve metal–metal bonding within fullerene structures, encouraging further investigation into the stabilization of metal–metal bonds with metals that typically resist bond formation. It is also reasonable to assume that compounds containing these novel single-electron metal–metal bonds could exhibit distinctive molecular magnetic properties, potentially leading to their applications in the field of molecular magnets as well as quantum information processing.

## Supporting Information Available

Experimental details, HPLC separation of CaY@*C*_*s*_(6)-C_82_ and CaY@*C*_2*v*_(5)-C_80_, crystallographic information, computational results, and pulse EPR results (PDF)
