# Supplementary material for: CaY@C2n: Exploring Molecular Qubits with Ca–Y Metal–Metal Bonds
Source: J Am Chem Soc. 2024 Aug 21;146(35):24310–9. doi: 10.1021/jacs.4c04720 (PMC11555672; doi:10.1021/jacs.4c04720)
Supplement: Supplementary file 1 — ja4c04720_si_001.pdf [file ja4c04720_si_001.pdf]

Supporting Information

CaY@C_{2n}: Exploring Molecular Qubits with Ca-Y Metal-Metal Bond

Jiawei Qiu[a, †], Laura Abella[b, †], Xiya Du[c, d, †], Zhengkai Cao[a], Zhiwen He[a], Qingyu Meng[a], Yingjing Yan[a], Josep M. Poblet[b], Lei Sun[c, d, e *], Antonio Rodríguez-Fortea[b, *], and Ning Chen[a, *]

[a] College of Chemistry, Chemical Engineering and Materials Science, and State Key Laboratory of Radiation Medicine and Protection, Soochow University, Suzhou, Jiangsu 215123, P.R. China.

[b] Departament de Química Física i Inorgànica. Universitat Rovira i Virgili, Marcel·lí Domingo 1, 43007 Tarragona, Spain.

[c] Department of Chemistry, School of Science and Research Center for Industries of the Future, Westlake University, Hangzhou, Zhejiang Province 310030, China.

[d] Institute of Natural Sciences, Westlake Institute for Advanced Study, Hangzhou, Zhejiang Province 310024, China.

[e] Key Laboratory for Quantum Materials of Zhejiang Province, Department of Physics, School of Science, Westlake University, Hangzhou, Zhejiang Province 310030, China.

†Jiawei Qiu, Laura Abella and Xiya Du contributed equally to this work.

* E-mail: chenning@suda.edu.cn

* E-mail: antonio.rodriguezf@urv.cat

* E-mail: sunlei@westlake.edu.cn

Table of Contents

Experimental Details 4

Figure S1. HPLC separation of CaY@C_s(6)-C_{82} 10

Figure S2. HPLC separation of CaY@C_{2v}(5)-C_{80} 12

Figure S3. HPLC chromatogram of purified CaY@C_s(6)-C_{82} and CaY@C_{2v}(5)-C_{80} 13

Figure S4. Disordered sites of CaY@C_s(6)-C_{82} 14

Figure S5. UV-vis-NIR absorption spectrum of CaY@C_s(6)-C_{82} and CaY@C_{2v}(5)-C_{80} 14

Table S1. Atom occupancy of metal sites in CaY@C_s(6)-C_{82} 15

Table S2. Crystallographic information of CaY@C_{82}·[Ni^{II}(OEP)]·2C_6H_6 15

Figure S6. DFT-optimized geometry of CaY@C_s(6)-C_{82} cage..................................16

Figure S7. DFT-optimized geometry of CaY@C_{2v}(5)-C_{80} cage 17

Table S3. Relative energies and Ca-Y distances for different orientations of the Ca and Y metal atoms inside the C_{2v}(5)-C_{80} cage 17

Figure S8. Different orientations of Ca and Y atoms within C_{2v}(5)-C_{80} 18

Table S4. Relative energies and Ca-Y distances for different orientations of the Ca and Y metal atoms inside the C_s(6)-C_{82} cage. 18

Figure S9. Different orientations of Ca and Y metal within C_s(6)-C_{82} 19

Figure S10. The potential electrostatic maps of C_{2v}(5)-C_{80}^{4-} and Isosurfaces (± 0.03 au) of the LUMO and LUMO+1 for C_{2v}(5)-C_{80}..19

Figure S11. The potential electrostatic maps of C_s(6)-C_{82}^{4-} and Isosurfaces (± 0.03 au) of the LUMO and LUMO+1 for C_s(6)-C_{82} 20

Figure S12. Molecular orbital (MO) diagrams for CaY@C_{2v}(5)-C_{80} and CaY@C_s(6)-C_{82}...20

Figure S13. Representation of the correlation between the hyperfine coupling constant and the Mulliken spin density of Y 21

Figure S14. Different X-ray orientations of Ca and Y metal atoms within the C_s(6)-C_{82} cage with their corresponding DFT-optimized structures. 21

Figure S15. Spin density distributions for the oxidized CaY@C_s(6)-C_{82}^{+} and reduced CaY@C_s(6)-C_{82}^{-} (triplet states) with an isosurface of ±0.002 a.u 22

xzy Coordinates 23

Figure S16. The X-band CW-EPR spectra of CaY@C_s(6)-C_{82} and CaY@C_{2v}(5)-C_{80} at 290 K along with the fitting results 27

Figure S17. Inversion recovery curves and Hahn echo decay curves of CaY@C_s(6)-C_{82} 28

Figure S18. Inversion recovery curves of CaY@C_s(6)-C_{82} via fitting with a mono-exponential decay 28

Figure S19. Hahn echo decay curves of CaY@C_s(6)-C_{82} via fitting with stretched exponential decay 29

Figure S20. Two-dimensional CP-ESEEM vs. τ_1 spectrum for CaY@C_s(6)-C_{82} 29

Figure S21. The frequency domain spectrum of Rabi oscillations after fast Fourier transformation 30

Table S5. Summary of spin−lattice relaxation time (T_1), spin decoherence time (T_2) and corresponding stretch factor q for CaY@C_s(6)-C_{82} 31

Figure S22. Comparison of the amount of Y_2@C_{82} and CaY@C_{82} 32

References 33

EXPERIMENTAL DETAILS

Synthesis and Isolation of CaY@C_{2n} (2n = 80 and 82). The carbon soot containing calcium and yttrium EMFs were produced by a modified Krätschmer-Huffman direct-current arc discharge method. The graphite rods, each of which was filled with 0.33 g of CaO, 0.67 g of Y_2O_3 and 2.13 g of graphite powder (molar ratio of Ca : Y : C = 1 : 1 : 30), were vaporized in the arcing chamber under 200 Torr He atmosphere. In total, about 300 graphite rods were burned. The carbon soot was collected and soaked in CS_2 solution for 24 h. CS_2 was removed by a rotary evaporator and then the resulting powder was redissolved in toluene for subsequent HPLC separation. The separation and purification of CaY@C_{2n} (2n = 80 and 82) were achieved by multistage HPLC procedures. Multiple HPLC columns, including Buckyprep M (25 mm × 250 mm, Cosmosil, Nacalai Tesque Inc.), Buckyprep (10 mm × 250 mm, Cosmosil, Nacalai Tesque, Japan), and 5PBB (10 mm × 250 mm, Cosmosil, Nacalai Tesque, Japan), were utilized in the procedures.

X-ray Crystallographic Study. Black block cocrystals of CaY@C_{82}·[Ni^{II}(OEP)]·2C_6H_6 were obtained by slow diffusion from a benzene solution of Ni^{II}(OEP) into a CS_2 solution of CaY@C_{82}. Single-crystal X-ray data of CaY@C_s(6)-C_{82} were collected at 173 K on a diffractometer (Bruker D8 Venture) equipped with a CCD collector. The multiscan method was used for absorption correction. The structures were solved using the intrinsic phasing methods and refined on F^2 using full-matrix least-squares using the SHELXL2018 crystallographic software packages. Hydrogen atoms were inserted at calculated positions and constrained with isotropic thermal parameters.

Spectroscopic Studies. The positive-ion mode matrix-assisted laser desorption/ionization time-of-flight (Bruker, Germany) was employed for mass characterization. The UV-vis-NIR absorption spectra of purified CaY@C_{2n} (2n = 80 and 82) were measured in CS_2 solution at room temperature with a Cary 5000 UV-vis-NIR spectrophotometer (Agilent, U.S.). The CW-EPR spectra of the purified CaY@C_{2n} (2n = 80 and 82) were measured at the X-band (ω =9.14 GHz) in CS_2 solution with a

JES-X320 spectrometer (JEOL, Japan).

X-band pulse EPR experiments. All X-band pulse EPR experiments were conducted on the CIQTEK EPR100 spectrometer equipped with 1.8 T bipolar Helmholtz electromagnet with high quality resonator, dielectric resonator and dual-mode resonator at the Instrumentation and Service Center for Molecular Sciences, Westlake University. A small amount of CaY@C_s(6)-C_{82} dissolved in toluene was vacuum sealed in 4 mm (external diameter) thick wall quartz tube frozen in liquid nitrogen. Before measurements, the magnetic field of instrument was calibrated by a standard DPPH sample.

For all pulse EPR measurements, π/2 and π pulses with lengths of 20 ns and 40 ns were chosen to maximize echo intensity besides Rabi oscillations. These pulse lengths were optimized by a three-pulse nutation sequence (nutation pulse − t − π/2 − τ − π − τ − echo), and the microwave attenuation was changed to match the pulse lengths making the first trough position at 40 ns at nutation curve between the intensity of the echo and the length of the nutation pulse. The shot repetition time (SRT) in all experiments was set to be longer than $5T_1$ to ensure that all spins have enough time to return to the thermal equilibrium state. To reduce the influence of noise as much as possible, the threshold for the ends of the echo integrator gate was set about 1/3 of echo amplitude from the baseline.

The echo-detected field sweep (EDFS) spectrum was obtained with the Hahn echo sequence (π/2 − τ− π − τ − echo) where τ was 200 ns by changing the magnetic field with 512 data points, 2048 shots per point and sweeps twice at 90 K. In this experiment, two-step phase cycling (+x, +x; -x, +x) was used to cancel background drift and defense pulse. Integration of the echo was plotted against the magnetic field, giving an EDFS spectrum. The collected EDFS spectrum was fitted using Easyspin on MATLAB R2023a.

The spin−lattice relaxation time (T_1) was measured by employing the inversion recovery sequence (π − t − π/2 − τ − π − τ − echo) where τ was fixed at 200 ns with 512 data points at the magnetic field with the maximum EDFS intensity (approximately 354

mT). The first length of t was 11 μs and the increment was adjusted to make the flat end of the curve accounts for 1/3 - 1/4 of the entire curve at variable temperature from 10 K to 170 K. In order to cancel background drift, defense pulse and other interference echoes, four-step phase cycling (+x, +x, +x; +x, -x, +x; -x, +x, +x; -x, -x, +x) was conducted. Integration of the echo was plotted against t, giving the inversion recovery curve. The obtained curves were fitted by a mono-exponential decay function:

$$I = ae^{-\frac{t}{T_1}} + I_0$$

Where I is echo intensity, a is a pre-factor, and I_0 represents baseline drift.

The spin decoherence time (T_2) was characterized by the Hahn echo sequence ($\pi/2 - \tau - \pi - \tau -$ echo) with 512 data points at the magnetic field with the maximum EDFS intensity (approximately 354 mT). τ stared at 200 ns and the increment was adjusted to make the flat end of the curve accounts for 1/3 - 1/4 of the entire curve at variable temperature from 10 K to 170 K. In order to cancel background drift and defense pulse, two-step phase cycling (+x, +x; -x, +x) was employed. Integration of the echo was plotted against 2τ, giving the Hahn echo decay curve. The obtained curves were fitted by a stretched exponential decay function:

$$I = ae^{-(\frac{2\tau}{T_2})^q} + I_0$$

Where I is echo intensity, a is a pre-factor, q is the stretch factor, and I_0 represents baseline drift.

The Rabi oscillations were implemented by a three-pulse nutation sequence (nutation pulse $- t - \pi/2 - \tau - \pi - \tau -$ echo) with 1024 data points at the magnetic field with the maximum EDFS intensity (approximately 354 mT) and temperature of 30 K. Here the delay time t was set as 40 μs which is greater than $5T_2$ and τ was 300 ns. The first length of nutation pulse was 10 ns and the increment was 1 ns. In order to cancel background drift, defense pulse and other interference echoes, four-step phase cycling (+x, +x, +x; +x, -x, +x; -x, +x, +x; -x, -x, +x) was conducted. Different Rabi oscillations curves were measured by changing the microwave attenuations (0, 3, 6, 9, 12, 15 dB) where microwave power was matched by varying pulse length. Integration of the echo

was plotted against the length of nutation pulse, giving Rabi oscillations curves. The obtained nutation curves were transformed to the frequency domain spectrum by subtracting baseline with linear fitting, processing with the Hamming window function, filling zeroes and performing fast Fourier transform (FFT) with OriginPro 2021. The observed peak frequency was assigned as the Rabi frequency. The Rabi frequencies were plotted with $10^{-\frac{A}{20\,dB}}$ (A represents the microwave attenuations in the unit of dB), which reflects the ratio between the magnetic field of the output microwave ($B_{MW\ output}$) and the input microwave ($B_{MW\ input}$).

The combination-peak electron spin echo envelope modulation (CP-ESEEM) vs. τ_1 experiment was conducted by seven-pulse sequence ($\pi/2 - \tau_1 - \pi/2 - t - \pi - t - \pi/2 - \tau_1 - \pi/2 - t_s - \pi/2 - \tau_2 - \pi - \tau_2 -$ echo) where the sequence of $\pi/2 - t_s - \pi/2 - \tau_2 - \pi - \tau_2 -$ echo as remote echo detection was used to measure echo modulation for τ_1 values shorter than the dead time.[1] The t_s and τ_2 were fixed at 8040 ns and 300 ns, respectively. τ_1 started at 80 ns and was incremented by 4 ns per step till 200 ns. For each τ_1, t started at 400 ns and was incremented by 8 ns with 512 data points. This experiment was carried out with 512 shots per point and at the magnetic field with the maximum EDFS intensity (approximately 354 mT) and temperature of 30 K. In order to cancel background drift, defense pulse and other interference echoes, eight-step phase cycling (+x, +x, +x, +x, +x, +x, +x; -x, +x, +x, +x, +x, +x, +x; +x, -x, +x, +x, +x, +x, +x; -x, -x, +x, +x, +x, +x, +x; +x, +x, +x, -x, +x, +x, +x; -x, +x, +x, -x, +x, +x, +x; +x, -x, +x, -x, +x, +x, +x; -x, -x, +x, -x, +x, +x, +x) was conducted. For each τ_1, integration of the echo was plotted against the t, giving time-domain CP-ESEEM spectrum. The obtained time-domain CP-ESEEM spectrum was transformed to the frequency-domain spectrum by subtracting baseline with exponential decay fitting, processing with the Hamming window function, filling zeroes and performing fast Fourier transform (FFT) by CIQTEK EPR100 software. The frequency-domain CP-ESEEM spectrum was plotted against τ_1, giving a two-dimensional CP-ESEEM spectrum.

The HYSCORE experiment was performed by seven-pulse sequence ($\pi/2 - \tau_1 - \pi/2 - t_1 - \pi - t_2 - \pi/2 - \tau_1 - \pi/2 - t_s - \pi/2 - \tau_2 - \pi - \tau_2 -$ echo) where the sequence of

$\pi/2 - t_s - \pi/2 - \tau_2 - \pi - \tau_2$ – echo as remote echo detection was used to measure echo modulation. The t_s and τ_2 were fixed at 8040 ns and 300 ns, respectively. τ_1 was fixed at 164 ns, which is the optimal delay time obtained by CP-ESEEM vs. τ_1. Both t_1 and t_2 started at 400 ns and were incremented 16 ns per step independently with 128 date points. This experiment was carried out with 400 shots per point and at the magnetic field with the maximum EDFS intensity (approximately 354 mT) and temperature of 30 K. In order to cancel background drift, the defense pulse and other interference echoes, eight-step phase cycling (+x, +x, +x, +x, +x, +x, +x; -x, +x, +x, +x, +x, +x, +x; +x, -x, +x, +x, +x, +x, +x; -x, -x, +x, +x, +x, +x, +x; +x, +x, +x, -x, +x, +x, +x; -x, +x, +x, -x, +x, +x, +x; +x, -x, +x, -x, +x, +x, +x; -x, -x, +x, -x, +x, +x, +x) was conducted. For each combination of t_1 and t_2, integration of the echo was plotted against the t_1 and t_2, giving time-domain HYSCORE spectrum. The obtained HYSCORE spectrum was transformed to frequency-domain spectrum by subtracting baseline with polynomial fitting, processing with the Hamming window function, filling zero and performing two-dimensional fast Fourier transform (FFT) in two dimensions by CIQTEK EPR100 software.

Computational Details. Kohn-Sham density functional theory (DFT) calculations were performed with the Amsterdam Density Functional (ADF, v. 2019) package.[2] The PBE0 exchange correlation functional[3-5] was used in conjunction with all-electron triple-ζ polarized (TZP) Slater-type orbital (STO) basis sets quality.[6, 7] Scalar relativistic (SR) zero-order regular approximation (ZORA) was included for relativistic effects.[8] 'D3' dispersion corrections by Grimme were also considered.[9, 10] ESR calculations were performed using an all-electron basis set with ADF. Calculations of redox properties were performed using the BP86 functional[11] and introducing solvent effects by means of the Conductor like Screening Model (COSMO),[12] as implemented in ADF using o-dichlorobenzene as a solvent.[13]

High performance liquid chromatography (HPLC) separation process of CaY@C_s(6)-C_{82}. Toluene was used as mobile phase in the multiple-step HPLC procedure. The first step was performed on a Buckyprep-M column (25 mm × 250 mm, Cosmosil Nacalai Tesque) (Figure S1 (a)). The fraction from 18 to 23.5 min (marked in red) containing CaY@C_{82} was collected and then injected into the Buckyprep column (10 mm × 250 mm, Cosmosil, Nacalai Tesque) for the second step. In the second step, the fraction from 46 to 52 min (marked in orange) was collected for the next step of HPLC separation (Figure S1 (b)). The third step was conducted on a 5PBB column (10 mm × 250 mm, Cosmosil, Nacalai Tesque) and the fraction from 87 to 96 min (marked in green) was collected (Figure S1 (c)). Then, this fraction was injected into a Buckyprep column (10 mm × 250 mm, Cosmosil, Nacalai Tesque) in recycle mode for the fourth step (Figure S1 (d)). The fraction marked in blue was collected and re-injected into the Buckyprep column (10 mm × 250 mm, Cosmosil, Nacalai Tesque) in recycle mode for the fifth step. After that, the fraction marked in pink, namely pure CaY@C_{82}, was finally obtained (Figure S1 (e)). Figure S1 (f) shows the purity of the isolated CaY@C_{82}.

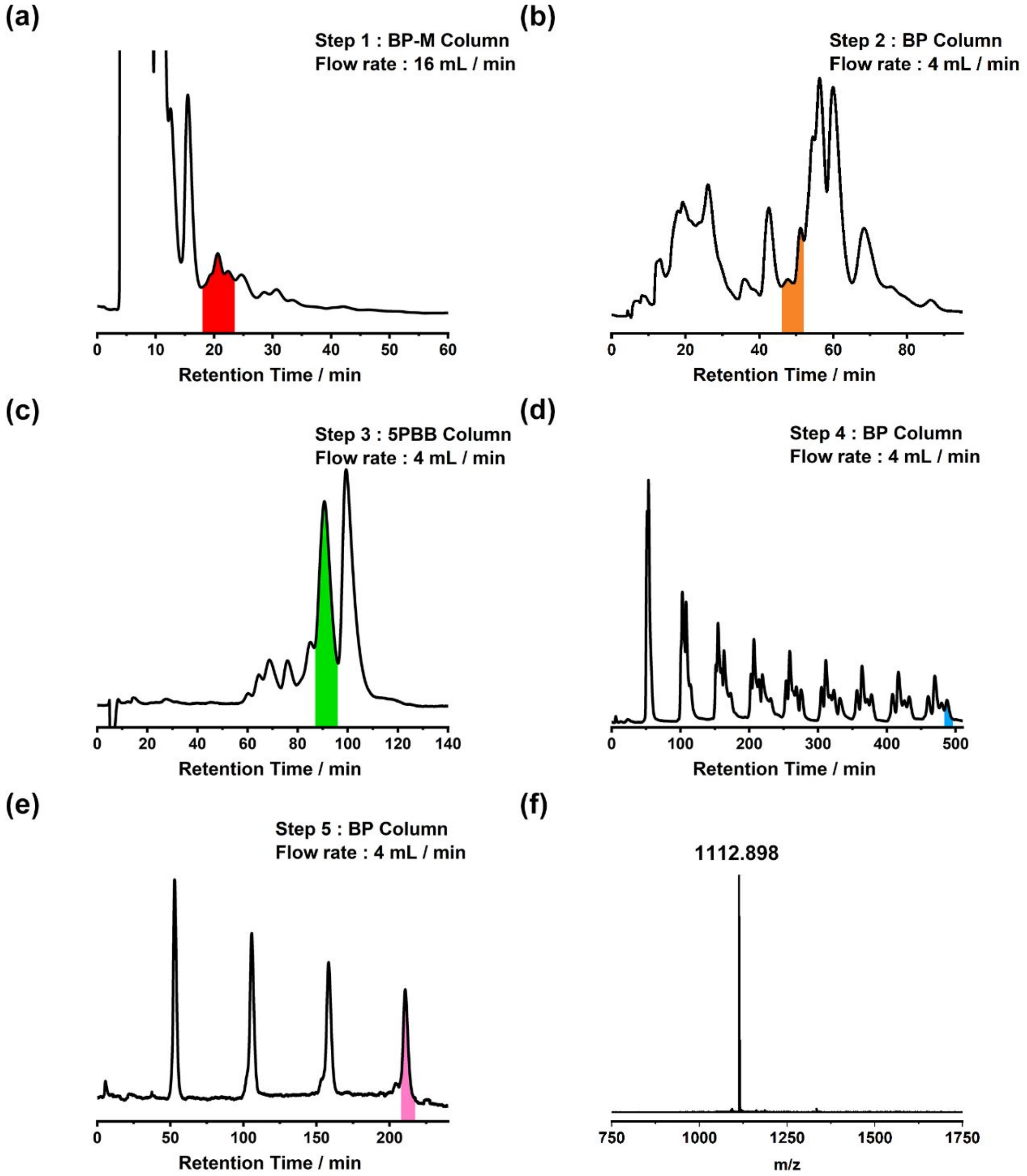

Figure S1. (a-e) HPLC profiles showing the isolation procedures of CaY@C_{82}. (f) The MALDI-TOF mass spectrum of the purified CaY@C_{82}.

High performance liquid chromatography (HPLC) separation process of CaY@C_{2v}(5)-C_{80} Toluene was used as mobile phase in the multiple-step HPLC procedure. The first step was performed on a Buckyprep-M column (25 mm × 250 mm, Cosmosil Nacalai Tesque) (Figure S2 (a)). The fraction from 18 to 23.5 min (marked in red) containing CaY@C_{80} was collected and then injected into the Buckyprep column (10 mm × 250 mm, Cosmosil, Nacalai Tesque) for the second step. In the second step, the fraction from 46 to 52 min (marked in orange) was collected for the next step of HPLC separation (Figure S2 (b)). The third step was conducted on a 5PBB column (10 mm × 250 mm, Cosmosil, Nacalai Tesque) and the fraction from 73 to 79 min (marked in green) was collected (Figure S2 (c)). Then, this fraction was injected into a Buckyprep column (10 mm × 250 mm, Cosmosil, Nacalai Tesque) in recycle mode for the fourth step. After that, the fraction marked in blue, namely pure CaY@C_{80}, was finally obtained (Figure S2 (d)). Figure S2 (e) shows the purity of the isolated CaY@C_{80}.

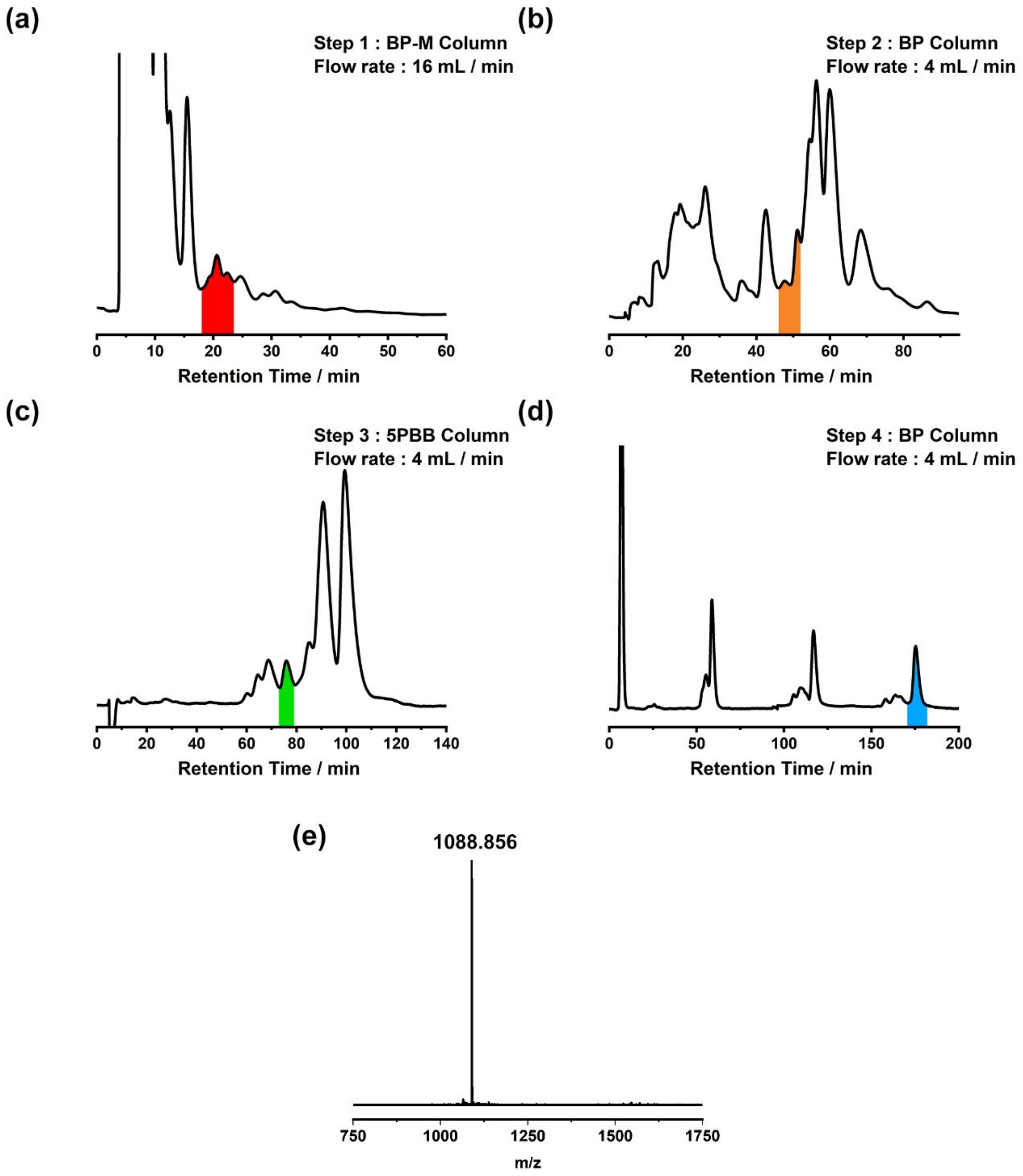

Figure S2. (a-d) HPLC profiles showing the isolation procedures of CaY@C_{80}. (e) The MALDI-TOF mass spectrum of the purified CaY@C_{80}.

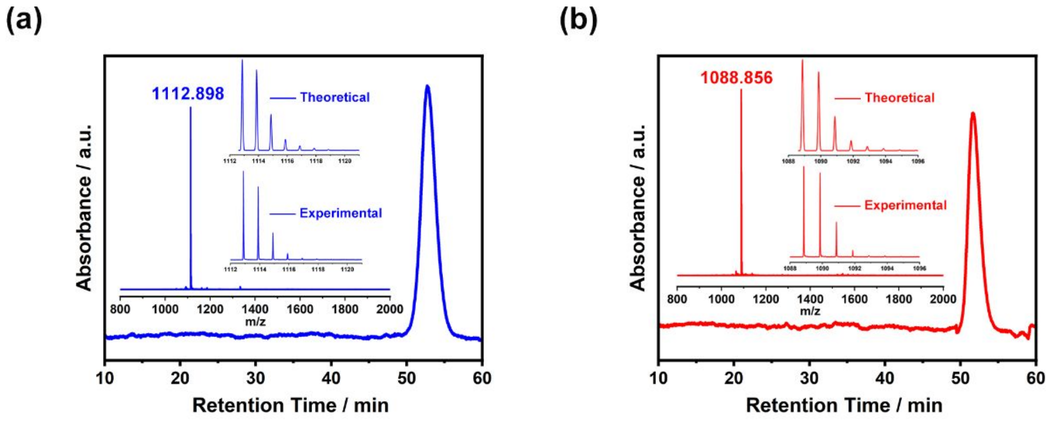

Figure S3. HPLC chromatogram of purified CaY@C_{82} (a) and CaY@C_{80} (b) on a Buckyprep column with toluene as the eluent (HPLC conditions: λ = 310 nm; flow rate = 4 mL/min). The insets show the positive-ion mode MALDI-TOF mass spectra and expansions of the corresponding experimental isotopic distribution of CaY@C_{82} (a) and CaY@C_{80} (b) in comparison with the theoretical one.

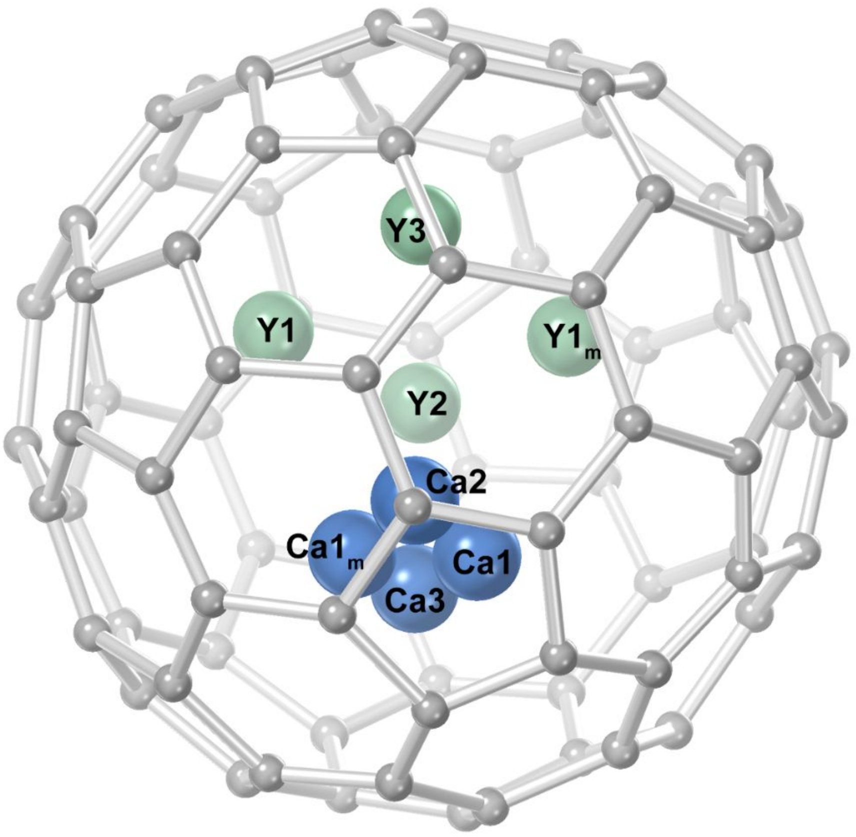

Figure S4. Drawing of all the Ca and Y disordered sites and their mirror-related counter-parts ($Ca1_m$ and $Y1_m$) in CaY@C_s(6)-C_{82}.

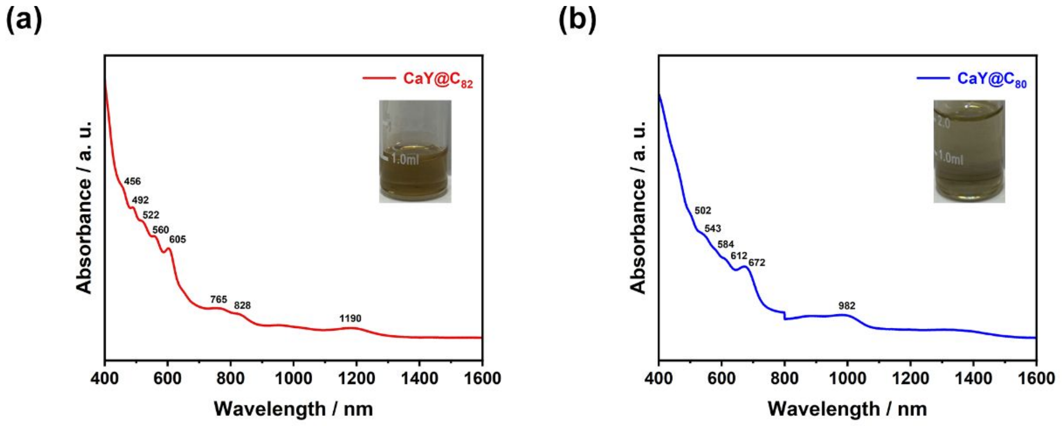

Figure S5. UV-vis-NIR absorption spectrum of CaY@C_{82} (a) and CaY@C_{80} (b). The inset shows the photograph of purified CaY@C_{82} (a) and CaY@C_{80} (b) dissolved in CS_2 solution.

Table S1. Atom occupancy of metal sites in CaY@C_s(6)-C_{82}.

Metal site	Ca1/Ca1$_m$	Y1/Y1$_m$	Ca2	Ca3	Y2	Y3
Occupancy	0.3157(17)	0.3157(17)	0.172(3)	0.196(3)	0.172(3)	0.196(3)

Table S2. Crystallographic information of CaY@C_{82}·[Ni^{II}(OEP)]·2C_6H_6

	CaY@C_{82}·[Ni^{II}(OEP)]·2C_6H_6
Empirical formula	$C_{130}H_{56}CaN_4NiY$
Formula weight	1861.48
Crystal system	monoclinic
Space group	*C*2/*m*
a, Å	25.2749(9)
b, Å	15.2299(5)
c, Å	19.9217(7)
α, deg	90
β, deg	95.247(2)
γ, deg	90
Volume, Å^3	7636.4(5)
Z	4
T, K	173(2)
F(000)	3804
ρ, g·cm^{-3}	1.619
θ, deg	2.227-77.942
R_1(all data)	0.1348
wR_2(all data)	0.2804
$R_1(I > 2.0\sigma(I))$	0.1069
$wR_2(I > 2.0\sigma(I))$	0.2589
parameters	1040
Goodness-of-fit indicator	1.083
R_{int}	0.0463

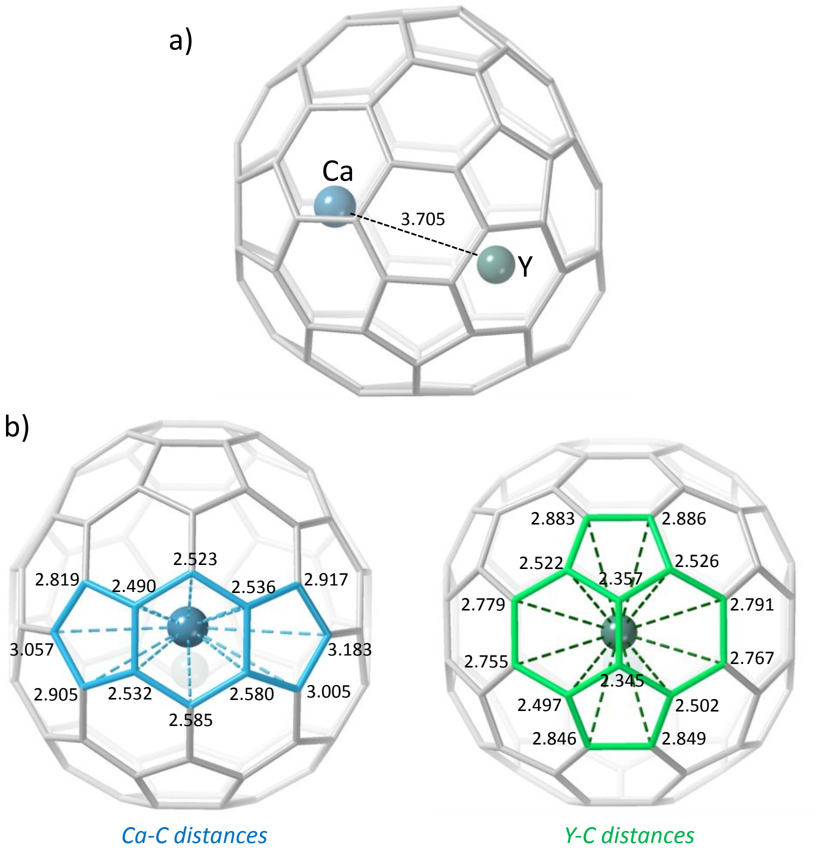

Figure S6. DFT-optimized geometry of CaY@C_s(6)-C_{82} cage. a) Ca-Y distance (in Å) is indicated. b) Ca-C and Y-C distances (in Å) are represented.

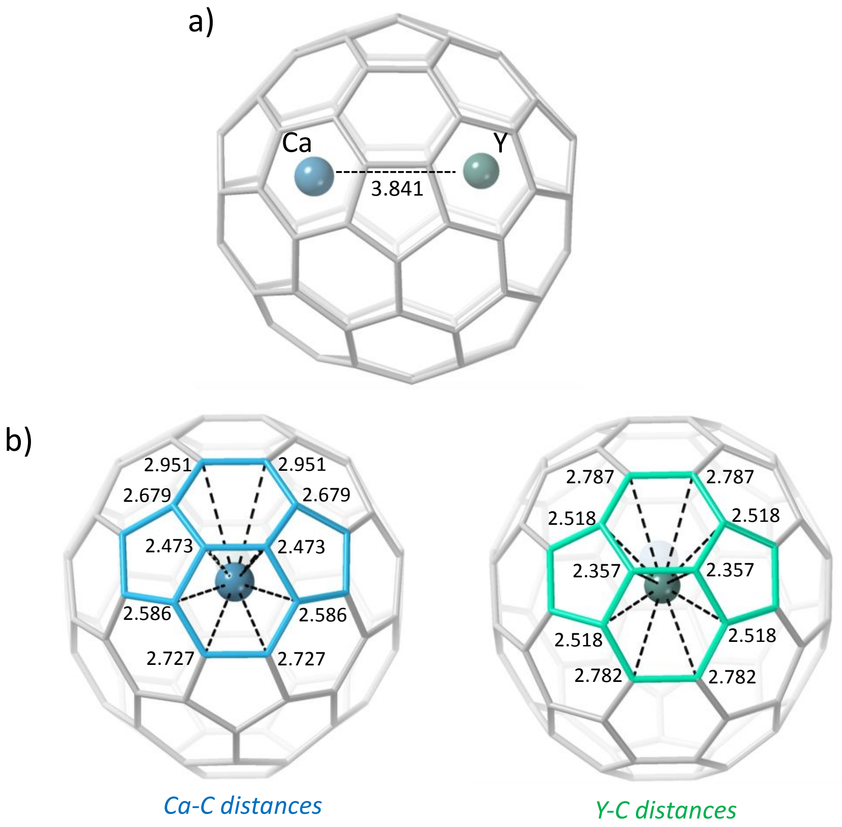

Figure S7. DFT-optimized geometry of CaY@C_{2v}(5)-C_{80} cage. a) Ca-Y distance (in Å) is indicated. b) Ca-C and Y-C distances (in Å) are represented.

Table S3. Relative energies (Erel, in kcal·mol^{-1}) and Ca-Y distances (in Å) for different orientations of the Ca and Y metal atoms inside the C_{2v}(5)-C_{80} cage. Orientations according to Figure S8.

Orientation	Erel	dist(Ca-Y)
**A**	0.0	3.841
**B**	4.2	3.866
**C**	12.2	3.543
**D**	21.8	3.982

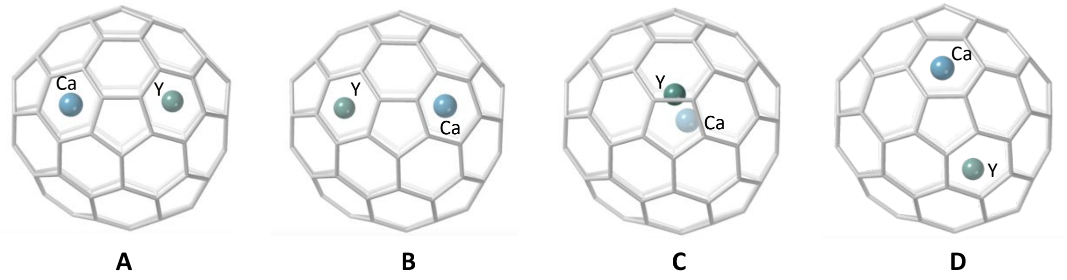

Figure S8. Different orientations of Ca and Y metal atoms within the $C_{2v}(5)$-C_{80} cage. Ca and Y are indicated in each DFT-optimized geometry.

Table S4. Relative energies (Erel, in kcal·mol^{-1}) and Ca-Y distances (in Å) for different orientations of the Ca and Y metal atoms inside the $C_s(6)$-C_{82} cage. Orientations according to Figure S9.

Orientation	Erel	dist(Ca-Y)
**A**	0.0	3.705
**B**	3.5	3.695
**C**	8.8	3.617
**D**	8.9	3.610
**E**	9.1	4.040
**F**	10.5	3.746
**G**	16.8	3.970
**H**	27.1	3.697
**I**	31.4	3.668

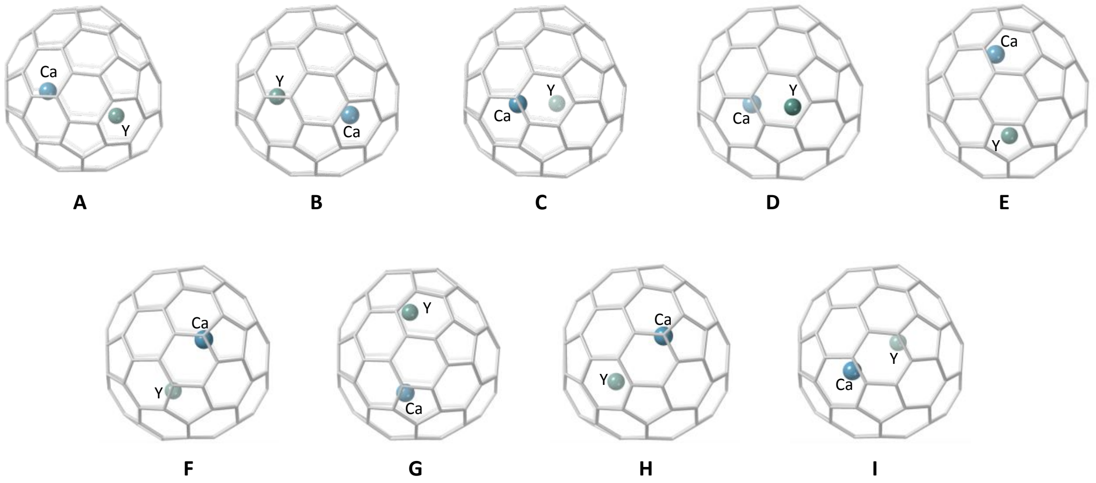

Figure S9. Different orientations of Ca and Y metal atoms within the $C_s(6)$-C_{82} cage. Ca and Y are indicated in each DFT-optimized geometry.

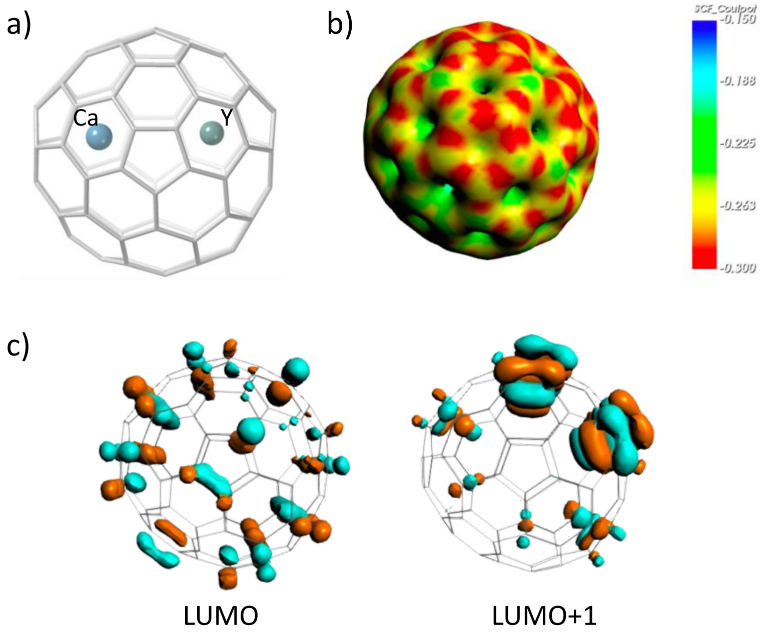

Figure S10. a) DFT-optimized geometry of CaY@$C_{2v}(5)$-C_{80} cage. b) Potential electrostatic map of $C_{2v}(5)$-C_{80}^{4-}. c) Isosurfaces ($\pm$0.03 au) of the LUMO and LUMO+1 for $C_{2v}(5)$-C_{80}.

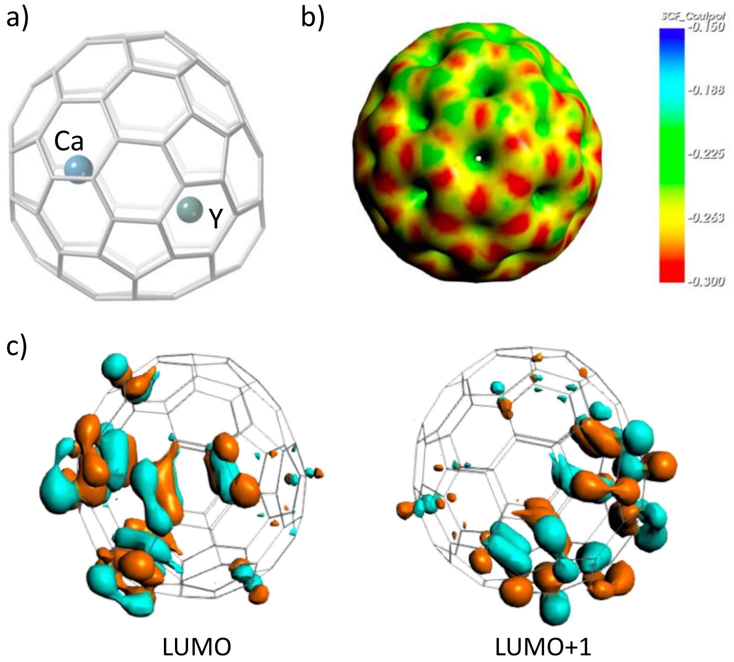

Figure S11. a) DFT-optimized geometry of CaY@C_s(6)-C_{82} cage. b) Potential electrostatic map of C_s(6)-C_{82}^{4-}. c) Isosurfaces (± 0.03 au) of the LUMO and LUMO+1 for C_s(6)-C_{82}.

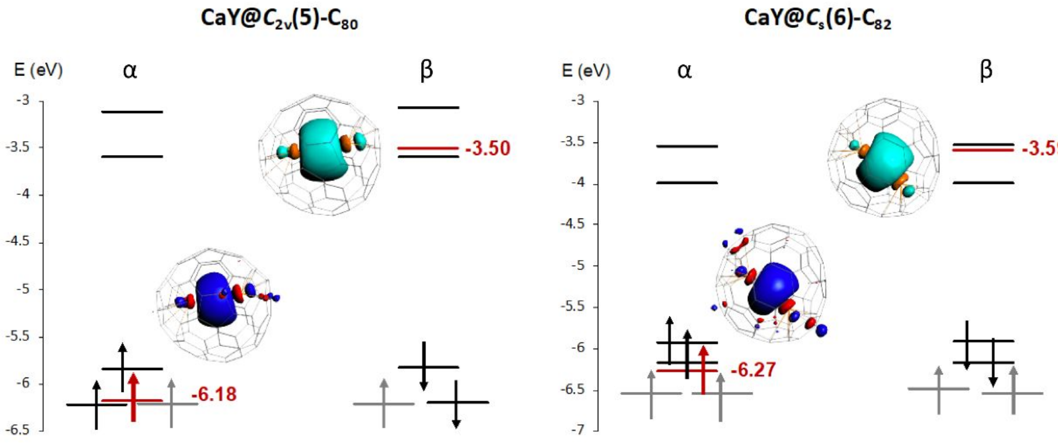

Figure S12. Molecular orbital (MO) diagrams for CaY@C_{2v}(5)-C_{80} (left) and CaY@C_s(6)-C_{82} (right). The energy levels of the delocalized sigma orbital a_1 (for alpha- and beta-spins) are drawn in red and the associated MO isosurfaces (±0.03 a.u.) with the corresponding MO energy (in eV) are shown on the side. The energy levels for the highest four electrons (in black) from the cage are also represented.

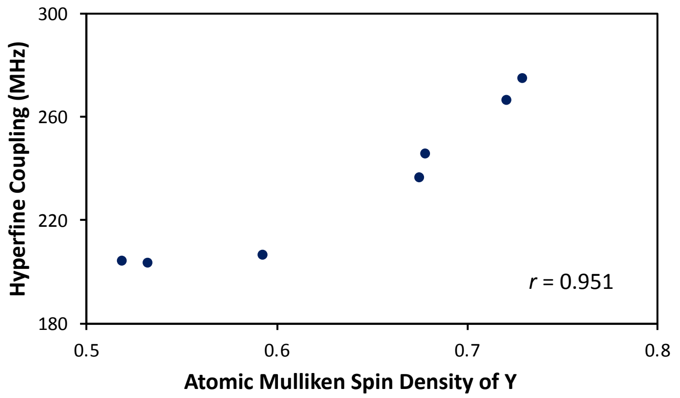

Figure S13. Representation of the correlation between the hyperfine coupling constant (in MHz) and the atomic Mulliken spin density of Y atom for all the X-Y@C_{2n} systems shown in Table 1. Correlation coefficient *r* is given inside the plot.

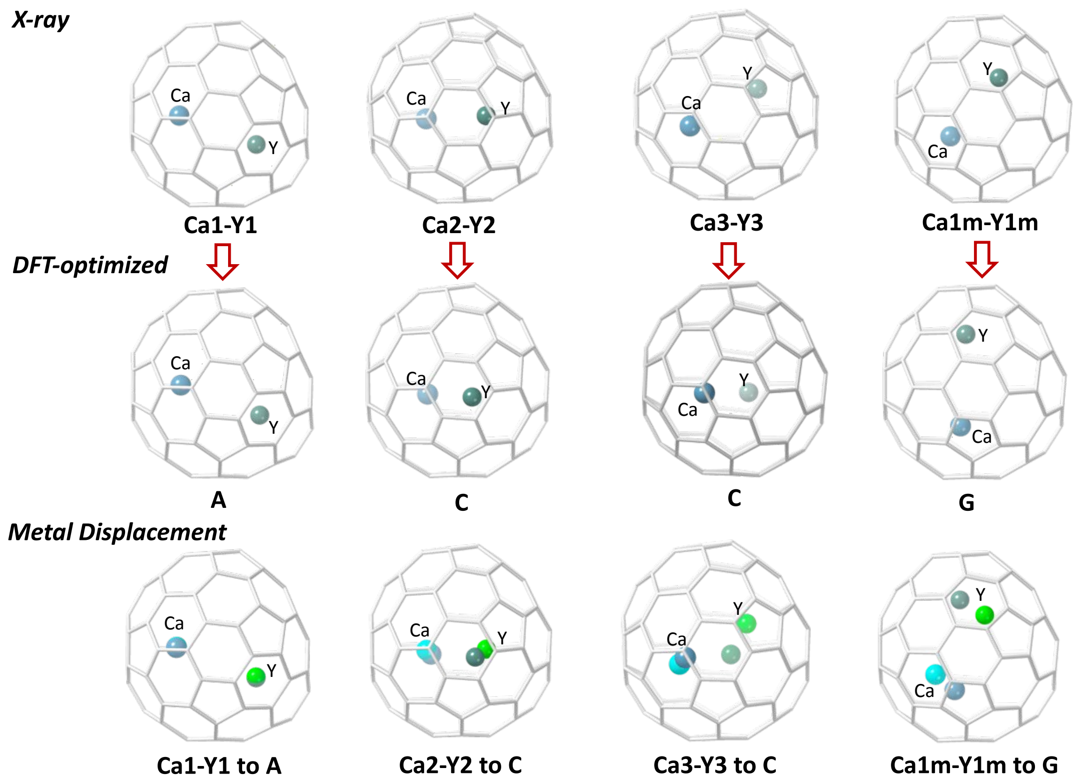

Figure S14. Different X-ray orientations of Ca and Y metal atoms within the C_s(6)-C_{82} cage with their corresponding DFT-optimized structures. The metal displacement during the DFT-optimization of Ca and Y is also represented. For both metals, lighter colour atoms correspond to the X-ray position and darker colour atoms to the DFT-optimized position. Ca and Y are indicated in each geometry.

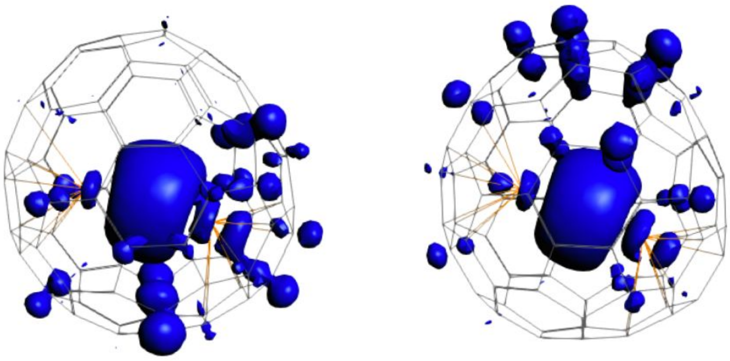

CaY@C_s(6)-C_{82}^{+} CaY@C_s(6)-C_{82}^{-}

Figure S15. Spin density distributions for the oxidized CaY@C_s(6)-C_{82}^{+} and reduced CaY@C_s(6)-C_{82}^{-} (triplet states) with an isosurface of ±0.002 a.u. Both oxidation and reduction are predicted to take place on the carbon cage and not in the single-electron occupied Ca-Y bonding orbital. The closed-shell singlet states are 6 kcal mol^{-1} higher in energy than the triplets for both the oxidized CaY@C_s(6)-C_{82}^{+} and reduced CaY@C_s(6)-C_{82}^{-} systems. The first oxidation potential (in o-dcb, +0.40 V) and the first reduction potential (-0.89 V), as well as the electrochemical gap (1.29 V), are comparable to those computed for Sc_2O@C_s(6)-C_{82} (1.18 V) and Sc_2S@C_s(6)-C_{82} (1.23 V), which share the same cage.[14] Therefore, the unpaired electron in the sigma bond seems to be rather well protected by the carbon cage.

xzy Coordinates

CaY@C_{2v}(5)-C_{80}

C	-4.163482	-1.205912	-0.711684
C	-3.411532	-2.227526	-1.422541
C	-2.772000	-1.623366	-2.560864
C	-3.177380	-0.242299	-2.592815
C	-3.977031	0.033389	-1.416209
C	-4.163482	-1.205912	0.711684
C	-3.411532	-2.227526	1.422541
C	-2.714974	-3.226540	0.721950
C	-2.714974	-3.226540	-0.721950
C	-1.412592	-3.662321	-1.176088
C	-0.736780	-3.054999	-2.265974
C	-1.465438	-2.020830	-3.004311
C	-0.719879	-1.052699	-3.717144
C	-1.163776	0.311295	-3.825539
C	-2.358792	0.749744	-3.188826
C	-2.397608	2.063387	-2.635748
C	-3.133191	2.324075	-1.418711
C	-3.841328	1.294220	-0.735278
C	-3.841328	1.294220	0.735278
C	-3.977031	0.033389	1.416209
C	-3.177380	-0.242299	2.592815
C	-2.772000	-1.623366	2.560864
C	-1.465438	-2.020830	3.004311
C	-0.736780	-3.054999	2.265974
C	-1.412592	-3.662321	1.176088
C	-0.687326	-4.072779	0.000000
C	0.679419	-4.069919	0.000000
C	1.406074	-3.663285	-1.176307
C	0.738475	-3.058878	-2.268401
C	1.466765	-2.026868	-3.006010
C	0.714187	-1.054158	-3.715129
C	1.153526	0.302793	-3.817291
C	-0.007686	1.150655	-3.863435
C	-0.020160	2.411010	-3.207078
C	-1.232633	2.886238	-2.645247
C	-1.219835	3.662862	-1.416508
C	-2.410843	3.328969	-0.697973
C	-2.410843	3.328969	0.697973
C	-3.133191	2.324075	1.418711
C	-2.397608	2.063387	2.635748
C	-2.358792	0.749744	3.188826

C	-1.163776	0.311295	3.825539
C	-0.719879	-1.052699	3.717144
C	0.714187	-1.054158	3.715129
C	1.466765	-2.026868	3.006010
C	0.738475	-3.058878	2.268401
C	1.406074	-3.663285	1.176307
C	2.702320	-3.225571	0.724087
C	2.702320	-3.225571	-0.724087
C	3.375703	-2.229739	-1.415738
C	2.769012	-1.637641	-2.573639
C	3.182272	-0.259268	-2.612044
C	2.352830	0.722153	-3.182254
C	2.365465	2.026569	-2.580909
C	1.199683	2.845749	-2.584950
C	1.232720	3.683564	-1.407979
C	-0.017528	3.992291	-0.730837
C	-0.017528	3.992291	0.730837
C	-1.219835	3.662862	1.416508
C	-1.232633	2.886238	2.645247
C	-0.020160	2.411010	3.207078
C	-0.007686	1.150655	3.863435
C	1.153526	0.302793	3.817291
C	2.352830	0.722153	3.182254
C	3.182272	-0.259268	2.612044
C	2.769012	-1.637641	2.573639
C	3.375703	-2.229739	1.415738
C	4.097240	-1.203105	0.696206
C	4.097240	-1.203105	-0.696206
C	3.960512	0.029274	-1.415644
C	3.867168	1.273794	-0.734903
C	3.149469	2.344391	-1.411844
C	2.485449	3.437802	-0.722183
C	2.485449	3.437802	0.722183
C	1.232720	3.683564	1.407979
C	1.199683	2.845749	2.584950
C	2.365465	2.026569	2.580909
C	3.149469	2.344391	1.411844
C	3.867168	1.273794	0.734903
C	3.960512	0.029274	1.415644
Y	1.203762	1.596021	0.000000
Ca	-1.840370	-0.746000	0.000000

CaY@C_s(6)-C_{82}

C	15.811640	15.376804	7.951065
C	17.576780	15.964443	9.612394
C	17.691401	17.298605	9.115269
C	16.814676	17.676556	8.057705
C	15.913704	16.718788	7.467369
C	15.813539	17.033876	6.064645
C	15.668451	16.003035	5.091331
C	20.045944	15.810395	10.001735
C	20.118715	17.190014	9.669702
C	18.964886	17.920317	9.194396
C	19.395121	18.835052	8.177287
C	18.572102	19.107140	7.060925
C	17.250978	18.576138	7.034783
C	16.630472	18.177683	5.799507
C	17.312935	18.300217	4.555755
C	17.066612	17.343424	3.544892
C	16.260687	16.188570	3.814410
C	17.906540	15.565821	2.314702
C	22.336504	15.525943	9.167022
C	22.347174	16.895107	8.729131
C	21.258514	17.713196	8.983232
C	20.827776	18.678873	8.010070
C	21.478207	18.787476	6.755394
C	20.665741	19.228065	5.630419
C	19.232212	19.300497	5.799110
C	18.616014	18.906490	4.572816
C	19.658150	18.584822	3.623882
C	19.439707	17.482277	2.697580
C	18.127571	16.948557	2.637443
C	20.571779	16.590796	2.411369
C	23.539434	15.659033	7.159467
C	23.038635	16.990328	7.473713
C	22.613070	17.898014	6.475168
C	22.831879	17.488676	5.098975
C	22.048319	17.974773	3.979202
C	20.938829	18.867146	4.249752
C	21.827677	16.845179	3.095604
C	22.582525	15.723201	3.608102
C	23.194738	16.115513	4.828137
Y	20.408140	16.780642	5.179171
C	16.949423	12.673750	8.250024
C	17.187040	13.697107	9.236362

C	16.614609	15.022690	9.096227
C	15.562333	14.345897	6.977527
C	16.135878	13.022366	7.115886
C	16.497265	12.563899	5.795326
C	17.598713	11.687949	5.556080
C	18.244923	11.173181	6.713743
C	17.993036	11.739749	8.025090
C	19.258936	11.821550	8.729846
C	19.587168	12.960978	9.514568
C	18.507008	13.851092	9.804665
C	18.738220	15.238906	10.036967
C	15.547779	14.665224	5.572094
C	16.131622	13.570190	4.853672
C	16.843185	13.759508	3.632181
C	17.967957	12.874486	3.343710
C	18.340647	11.823917	4.302135
C	19.679335	11.348150	4.289387
C	20.324184	10.857534	5.481702
C	19.620157	10.779204	6.672022
C	20.246382	11.179474	7.917167
C	21.551586	11.643497	7.922804
C	21.912695	12.779023	8.733568
C	20.949549	13.495400	9.494013
C	21.176298	14.929399	9.726624
C	16.806528	15.068983	3.080699
C	18.973274	14.734022	2.016363
C	18.968485	13.368454	2.466497
C	20.346661	12.990921	2.620636
C	20.695038	12.005803	3.513625
C	21.897232	12.127524	4.306805
C	21.691275	11.337260	5.489713
C	22.288998	11.719566	6.678050
C	23.105789	12.901941	6.711511
C	22.967945	13.462480	8.036669
C	23.176357	14.803853	8.250882
C	20.314606	15.245561	2.054294
C	21.179520	14.146944	2.463727
C	22.262913	14.355167	3.335725
C	22.629492	13.338437	4.326620
C	23.261726	13.743464	5.584602
C	23.523291	15.162854	5.843726
Ca	18.127090	14.593037	7.111583

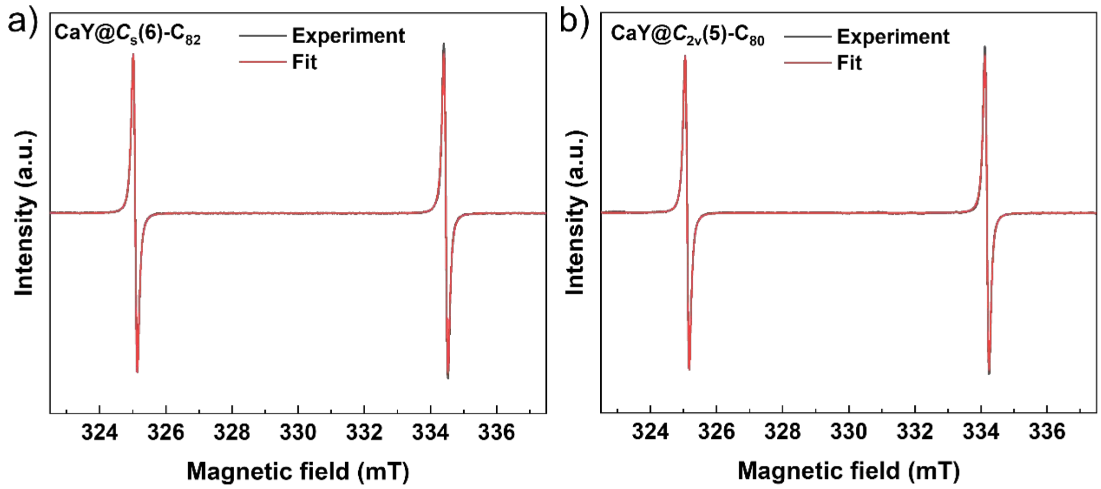

Figure S16. The X-band CW spectra of (a) CaY@C_s(6)-C_{82} and (b) CaY@C_{2v}(5)-C_{80} measured in CS_2 solution at 290 K. Each spectrum was fitted using Easyspin 6.0.0 in MATLAB R2024a with a S = 1/2 electron spin system coupled with a I = 1/2 nuclear spin. The spectrum lines were found to exhibit the Lorentzian lineshape. For CaY@C_s(6)-C_{82}, the fitted g_{iso}, A, and linewidth are 1.981855(2), 260.42(1) MHz, and 0.0989(3) mT, respectively. For CaY@C_{2v}(5)-C_{80}, the fitted g_{iso}, A and linewidth are 1.982660(2), 251.65(1) MHz, and 0.0994(3) mT, respectively.

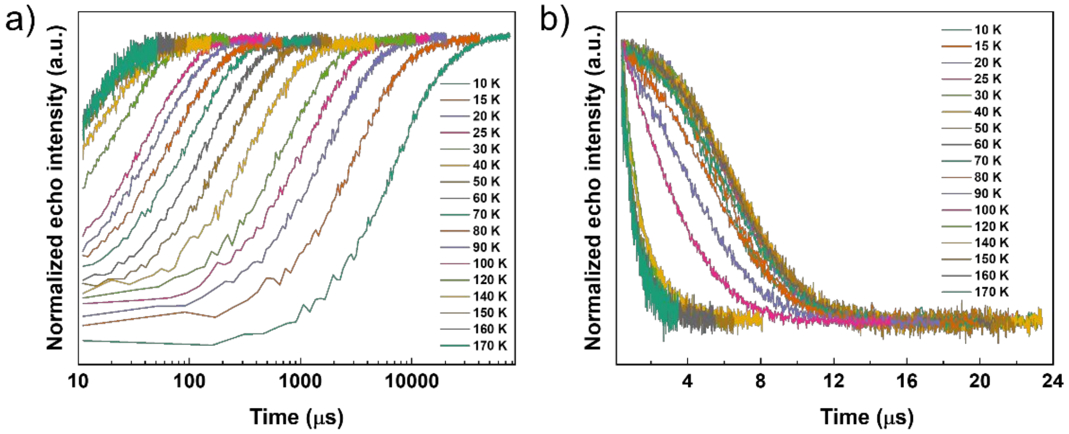

Figure S17. (a) Inversion recovery curves and (b) Hahn echo decay curves of CaY@C_s(6)-C_{82} dissolved in toluene at variable temperature from 10 K to 170 K.

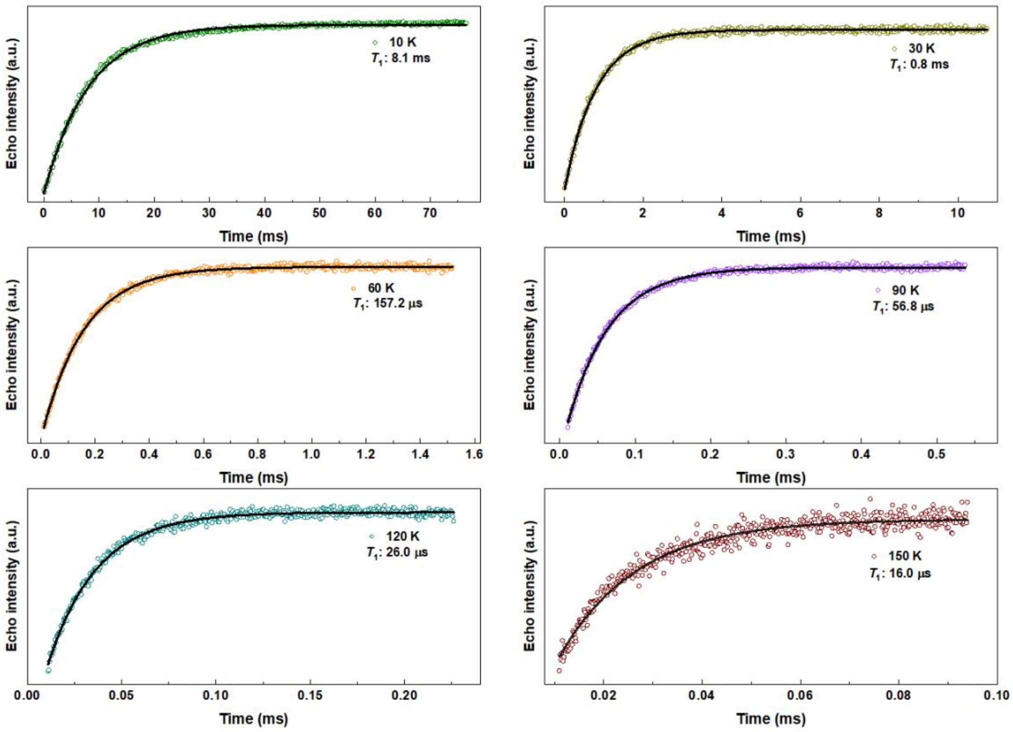

Figure S18. Inversion recovery curves of CaY@C_s(6)-C_{82} dissolved in toluene via fitting with a mono-exponential decay at 10, 30, 60, 90, 120, 150 K. The black lines are the fitting curves.

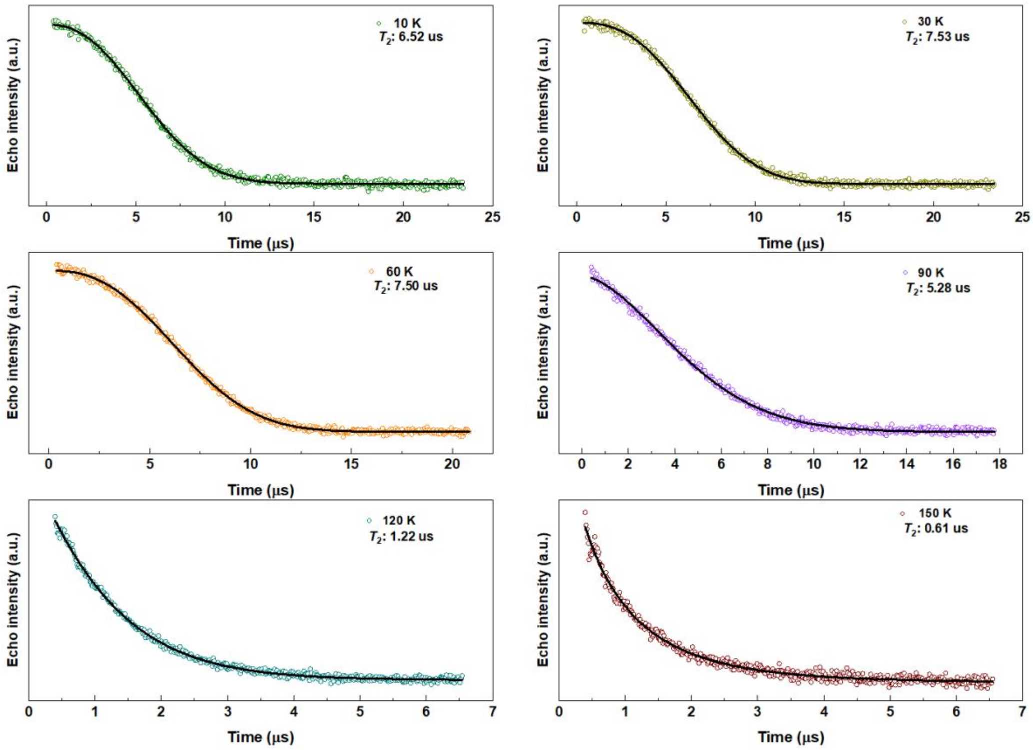

Figure S19. Hahn echo decay curves of CaY@C_s(6)-C_{82} dissolved in toluene via fitting with stretched exponential decay at 10, 30, 60, 90, 120, 150 K. The black lines are the fitting curves.

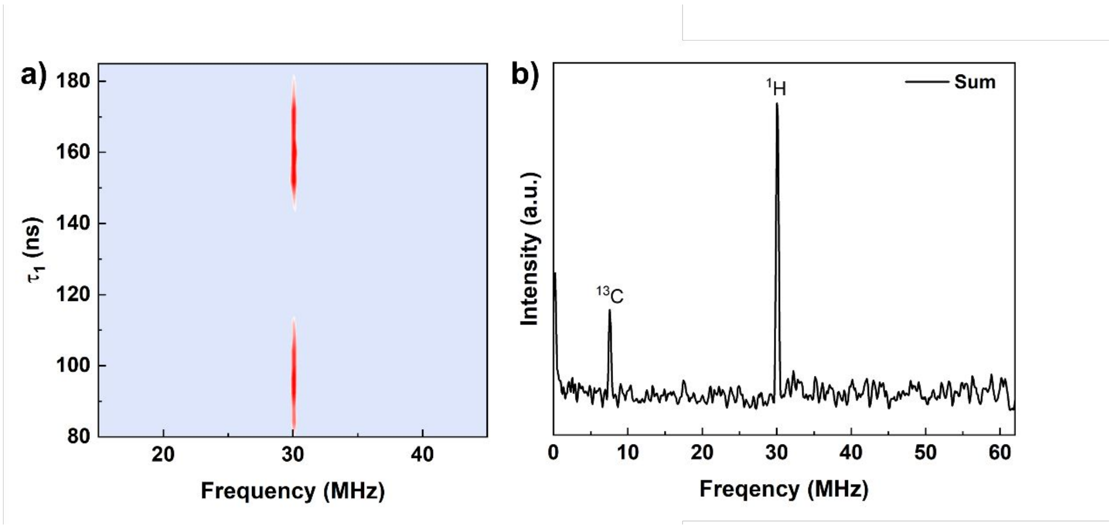

Figure S20. a) Two-dimensional CP-ESEEM vs. τ_1 spectrum and b) the corresponding superimposed intensity across all τ_1 values from 80 to 200 ns at 30 K for CaY@C_s(6)-C_{82} dissolved in toluene.

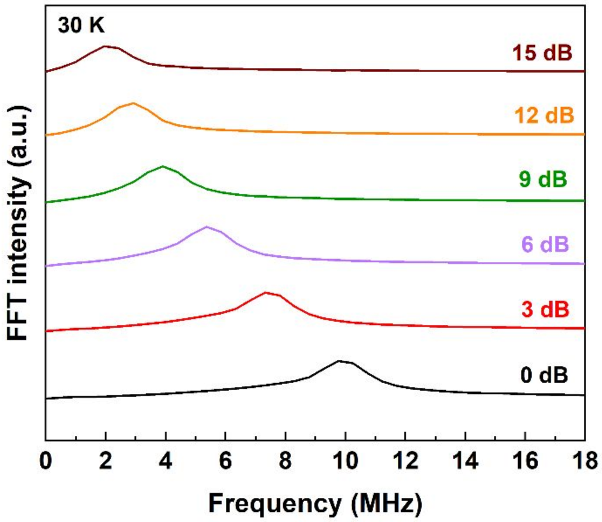

Figure S21. The frequency domain spectrum of Rabi oscillations after fast Fourier transformation at different microwave attenuations for CaY@C_s(6)-C_{82} dissolved in toluene.

Table S5. Summary of spin−lattice relaxation time (T_1), spin decoherence time (T_2) and corresponding stretch factor q at variable temperature from 10 K to 170 K for CaY@C_s(6)-C_{82} dissolved in toluene.

Temperature (K)	T_1 (μs)	T_2 (μs)	q
10	$8.093(40) \times 10^3$	6.52(2)	2.36(2)
15	$3.507(19) \times 10^3$	6.79(2)	2.49(3)
20	$1.929(10) \times 10^3$	7.25(2)	2.69(2)
25	$1.213 (6) \times 10^3$	7.37(1)	2.64(2)
30	$8.01(5) \times 10^2$	7.54(1)	2.67(2)
40	$4.01(3) \times 10^2$	7.74(2)	2.67(2)
50	$2.42(1) \times 10^2$	7.68(2)	2.61(2)
60	157.2(8)	7.50(1)	2.53(2)
70	108.2(5)	7.18(1)	2.38(2)
80	77.9(5)	6.49(2)	2.09(2)
90	56.8(2)	5.28(2)	1.73(2)
100	45.6(2)	3.66(2)	1.39(1)
120	26.0(2)	1.22(2)	1.12(2)
140	19.2(3)	0.74(4)	0.72(2)
150	16.0(3)	0.61(5)	0.74(3)
160	12.2(4)	0.69(6)	0.86(5)
170	9.5(4)	0.78(6)	1.07(9)

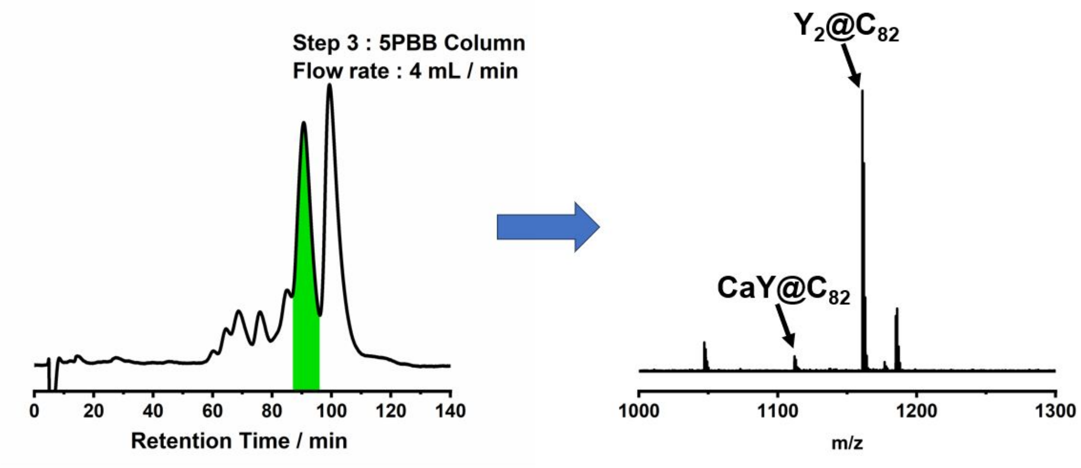

Figure S22. Comparison of the yield of $Y_2@C_{82}$ and $CaY@C_{82}$. The figure on the left shows the chromatogram of the third step of the separation of $CaY@C_{82}$ and the right one is the corresponding MALDI-TOF-MS spectrum. The yield of $CaY@C_{82}$ is estimated to be about 1/20 of that of the $Y_2@C_{82}$.

REFERENCES

(1) Cho, H.; Pfenninger, S.; Gemperle, C.; Schweiger, A.; Ernst, R. R. Zero deadtime pulsed ESR by remote echo detection. *Chem. Phys. Lett.* **1989**, *160* (4), 391-395.

(2) te Velde, G.; Bickelhaupt, F. M.; Baerends, E. J.; Fonseca Guerra, C.; van Gisbergen, S. J. A.; Snijders, J. G.; Ziegler, T. Chemistry with ADF. *J. Comput. Chem.* **2001**, *22* (9), 931-967.

(3) Perdew, J. P.; Burke, K.; Ernzerhof, M. Generalized Gradient Approximation Made Simple. *Phys. Rev. Lett.* **1996**, *77* (18), 3865-3868.

(4) Perdew, J. P.; Burke, K.; Ernzerhof, M. Generalized Gradient Approximation Made Simple [Phys. Rev. Lett. 77, 3865 (1996)]. *Phys. Rev. Lett.* **1997**, *78* (7), 1396-1396.

(5) Adamo, C.; Barone, V. Toward reliable density functional methods without adjustable parameters: The PBE0 model. *J. Chem. Phys.* **1999**, *110* (13), 6158-6170.

(6) Perdew, J. P. Density-functional approximation for the correlation energy of the inhomogeneous electron gas. *Phys. Rev. B.* **1986**, *33* (12), 8822-8824.

(7) Becke, A. D. Density functional calculations of molecular bond energies. *J. Chem. Phys.* **1986**, *84* (8), 4524-4529.

(8) Lenthe, E. v.; Baerends, E. J.; Snijders, J. G. Relativistic regular two-component Hamiltonians. *J. Chem. Phys.* **1993**, *99* (6), 4597-4610.

(9) Grimme, S.; Antony, J.; Ehrlich, S.; Krieg, H. A consistent and accurate ab initio parametrization of density functional dispersion correction (DFT-D) for the 94 elements H-Pu. *J. Chem. Phys.* **2010**, *132* (15), 154104.

(10) Grimme, S.; Ehrlich, S.; Goerigk, L. Effect of the damping function in dispersion corrected density functional theory. *J. Comput. Chem.* **2011**, *32* (7), 1456-1465.

(11) Becke, A. D. Density-functional exchange-energy approximation with correct asymptotic behavior. *Phys. Rev. A.* **1988**, *38* (6), 3098-3100.

(12) Klamt, A. Conductor-like Screening Model for Real Solvents: A New Approach to the Quantitative Calculation of Solvation Phenomena. *J. Phys. Chem.* **1995**, *99* (7), 2224-2235.

(13) Valencia, R.; Rodríguez-Fortea, A.; Clotet, A.; de Graaf, C.; Chaur, M. N.; Echegoyen, L.; Poblet, J. M. Electronic Structure and Redox Properties of Metal Nitride Endohedral Fullerenes $M_3N@C_{2n}$ (M=Sc, Y, La, and Gd; 2n=80, 84, 88, 92, 96). *Chem. Eur. J.* **2009**, *15* (41), 10997-11009.

(14) Mercado, B. Q.; Chen, N.; Rodríguez-Fortea, A.; Mackey, M. A.; Stevenson, S.; Echegoyen, L.;

Poblet, J. M.; Olmstead, M. M.; Balch, A. L. The Shape of the $Sc_2(\mu_2\text{-}S)$ Unit Trapped in C_{82}: Crystallographic, Computational, and Electrochemical Studies of the Isomers, $Sc_2(\mu_2\text{-}S)@C_s(6)\text{-}C_{82}$ and $Sc_2(\mu_2\text{-}S)@C_{3v}(8)\text{-}C_{82}$. *J. Am. Chem. Soc.* **2011**, *133* (17), 6752-6760.
